# Effects of inulin-type oligosaccharides (JSO) from *Cichorium intybus L*. on behavioral deficits induced by chronic restraint stress in mice and associated molecular alterations

**DOI:** 10.3389/fphar.2024.1484337

**Published:** 2024-11-01

**Authors:** Caihong Yao, Ning Jiang, Xinran Sun, Yiwen Zhang, Ruile Pan, Qinghu He, Qi Chang, Xinmin Liu

**Affiliations:** ^1^ Research Center for Pharmacology and Toxicology, Institute of Medicinal Plant Development (IMPLAD), Chinese Academy of Medical Sciences and Peking Union Medical College, Beijing, China; ^2^ College of Traditional Chinese Medicine, Hunan University of Chinese Medicine, Changsha, China; ^3^ Institute of Drug Discovery Technology, Ningbo University, Ningbo, China

**Keywords:** *Cichorium intybus L.*, inulin-type oligosaccharides(JSO), depression, anxiety, chronic restraint stress, molecular alterations

## Abstract

Depression and anxiety are serious psychiatric disorders with significant physical and mental health impacts, necessitating the development of safe and effective treatments. This study aimed to evaluate the efficacy of *Jiangshi* oligosaccharide (JSO), a type of inulin-based oligosaccharide, in alleviating anxiety and depression and to investigate the underlying molecular mechanisms. Using a mouse model of chronic restraint stress (CRS), JSO was administered orally at doses of 50, 100, and 200 mg/kg for 21 days. Behavioral tests, including the novelty-suppressed feeding test (NSFT), open field test (OFT), elevated plus maze test (EPMT), tail suspension test (TST), and forced swimming test (FST), demonstrated that JSO significantly improved anxiety- and depressive-like behaviors (P< 0.05). Notably, JSO reduced feeding latency in the NSFT, increased time spent in the center in the OFT, enhanced time and entries into open arms in the EPMT, and decreased immobility time in the TST and FST (P< 0.01). Histological and molecular analyses revealed that JSO treatment attenuated neuronal loss in the hippocampus (Hip) and medial prefrontal cortex (mPFC) and reduced the expression of inflammatory markers such as Iba-1 and GFAP in these regions. JSO significantly downregulated the mRNA and protein expression of pro-inflammatory factors (IL-1β, TNF-α, IL-6) while increasing anti-inflammatory markers (IL-10, TGF-β) (P< 0.05). Furthermore, JSO inhibited the c-GAS-STING-NLRP3 axis and apoptosis-related proteins (Bax/Bcl-2, Caspase-3/8/9) while promoting the expression of brain-derived neurotrophic factor (BDNF), PSD-95, and synaptophysin (SYP), indicating improved neuronal survival and synaptic plasticity (P< 0.01). These findings suggest that JSO exerts potent anti-anxiety and antidepressant effects by modulating neuroinflammation, synaptic function, and neuronal apoptosis in the Hip and mPFC of CRS mice. This study highlighted JSO as a potential therapeutic agent for stress-induced anxiety and depression.

## 1 Introduction

Anxiety and depression are common psychiatric disorders that can lead to multiple adverse symptoms, such as suicidal tendencies, sleep disorders, anhedonia, and cognitive dysfunction (Wang et al., 2018; Morel et al., 2022; Chen et al., 2023; Fu et al., 2023). Presently, most available clinical drugs only focus on anti-anxiety or anti-depression; drugs to improve depression/anxiety comorbidity are lacking (Chen et al., 2023). In addition, medications commonly used for improving anxiety/depression have serious side effects, such as nausea and vomiting (Shen et al., 2021). Conventional anti-depression/anxiety treatments have shown extreme limitations, including a delay in the onset and low efficacy in over 30% of patients (Li Y. et al., 2023). Thus, more efficacious medicines with fewer side effects are urgently needed.

Oligosaccharides, including inulin-type variants, have been extensively studied for their prebiotic effects, primarily influencing the gut microbiota and promoting gut-brain axis interactions. These interactions have been suggested to impact mood regulation, cognitive function, and stress resilience through modulation of gut bacteria and the production of short-chain fatty acids (SCFAs), which may exert neuroprotective effects (Arioz et al., 2019; Crowley et al., 2006). Inulin-type oligosaccharides function not only as prebiotics, but also potentially exhibit anti-inflammatory and antioxidant properties, which may contribute to their neuroprotective effects (Chen et al., 2023). Oligosaccharides are widely distributed across different plant species, including *Cichorium intybus L.* and *Smallanthus sonchifolius*, while their composition and biological activity can vary. For instance, inulin-type oligosaccharides isolated from *S. sonchifolius* (yacon) are shorter in chain length compared with those from *C. intybus L.*, which may affect their bioactivity (An et al., 2016). Such differences in oligosaccharide composition can influence their potential therapeutic effects, warranting further comparison across plant sources. Inulin-type oligosaccharides were extracted from *C. intybus L.* using a standardized extraction method, involving hot-water extraction, followed by ethanol precipitation, as previously described (Sun et al., 2019). This standardized method ensures consistency in the yield and composition of oligosaccharides, minimizing variability in experimental outcomes.

*Cichorium intybus L.*, a plant in the Asteraceae family, has attracted increasing attention due to its health-promoting properties and is an abundant source of oligosaccharides (Lightowler et al., 2018; Perović et al., 2021). Research has shown that the inulin-type oligosaccharides (molecule, GFn, n = 5–11) isolated from the plant *S. sonchifolius* may have anti-depressant effects in an acute experimental model (An et al., 2016). However, the potential therapeutic effects of inulin-type oligosaccharides on stress-induced behavioral deficits have not been reported. Considering these findings, we hypothesized that the inulin-type oligosaccharides (molecule, GFn, n = 4–20) (JSO) isolated from the plant *Cichorium intybus L.* may have potential benefits in a model of stress-induced behavioral deficits.

Chronic restraint stress (CRS) is a recognized model of psychosocial stress that symbolizes the development of relatively mild, predictable, yet inevitable chronic stress, and simulates chronic stress exposure in stressful work and limited living spaces (Wang et al., 2020; Kwatra et al., 2021). Studies have indicated that CRS can induce behavioral deficits similar to those observed in anxiety and depression (MacDowell et al., 2021; Zhou et al., 2021). Furthermore, CRS can induce neuronal loss and neuroplastic defects, microglial and astrocyte activation, inflammatory responses, and apoptosis in the brain, similar to the clinical symptoms of anxiety/depression (Liu et al., 2020; Song et al., 2020; Fan et al., 2023; Ma et al., 2023).

Although inulin-type oligosaccharides from various sources have been investigated for their health-promoting effects, evidence regarding their specific role in alleviating stress-induced behavioral deficits is still emerging. Previous studies, such as those conducted by Huo et al. (2017), have demonstrated promising results in related stress models, while more research is needed to confirm these effects and explore the underlying molecular mechanisms. Therefore, using the mouse model of CRS, this study aimed to evaluate the efficacy of JSO in ameliorating stress-induced behavioral deficits and to explore the associated molecular changes. The use of a standardized extraction method and dose range is critical to ensure reproducibility and comparability with previous studies. The oligosaccharides used in this study were prepared following a rigorous protocol to ensure the purity and consistency of the extracted compounds. The doses selected for this study were based on prior research exploring dose-dependent effects of similar oligosaccharides in stress models (Sun et al., 2019; Huo et al., 2017). Despite the growing evidence of the neuropsychiatric benefits of oligosaccharides, their therapeutic potential in ameliorating stress-induced anxiety and depression comorbidities remains unexplored. This study is the first to investigate the effects of JSO on CRS-induced behavioral deficits, positioning JSO as a novel candidate for addressing neuropsychiatric disorders. Furthermore, by examining the associated molecular changes, such as neuroinflammation, synaptic plasticity, and apoptosis, this research provided new insights into the mechanisms underlying the potential therapeutic benefits of JSO. The findings could pave the way for developing a more effective and safer treatment for anxiety and depression comorbidity.

## 2 Materials and methods

### 2.1 Animals

Male C57BL/6N mice, aged 7–8 weeks, were obtained from the Vital River Co., Ltd. (qualified no. SCXK 2021–0006, Beijing, China). In this study, mice were housed in groups of 4-5 per cage. This group size was chosen based on standard animal care guidelines to ensure social enrichment while minimizing stress and aggression. Mice were housed in cages at random under a 12-h light/dark cycle (lights on at 8:30 a.m., light off at 8:30 p.m.), with 55% ± 10% relative comfortable humidity and a pleasant temperature at 24°C ± 2°C. In this period, water and food were freely accessed. After 1 week of adaptation, the experiment began. All experimental stages were carried out in accordance with the Animal Ethics Committee of the Institute of Medicinal Plant Development, Peking Union Medical College (Approval No. SYXK 2023–0049).

### 2.2 Agents

Citalopram (Cit) is a well-known selective serotonin reuptake inhibitor (SSRI) used to treat depression and anxiety. The chosen dose of 10 mg/kg for citalopram in mice is supported by several studies that have demonstrated its efficacy in ameliorating anxiety and depression-like behaviors induced by chronic stress. For instance, Crowley et al. (2005) used a dose of 10 mg/kg citalopram in mice and observed significant antidepressant-like effects in the forced swim test, a commonly used assay for evaluating antidepressant activity. Burstein et al. (2014) also utilized 10 mg/kg citalopram in mice and reported a reduction in depressive-like behaviors in the tail suspension test. These studies indicated that 10 mg/kg is an effective dose for modulating mood-related behaviors in rodent models. Cit was obtained from Shanghai Macklin Biochemical Technology Co., Ltd. (Shanghai, China).

**FIGURE 1:**
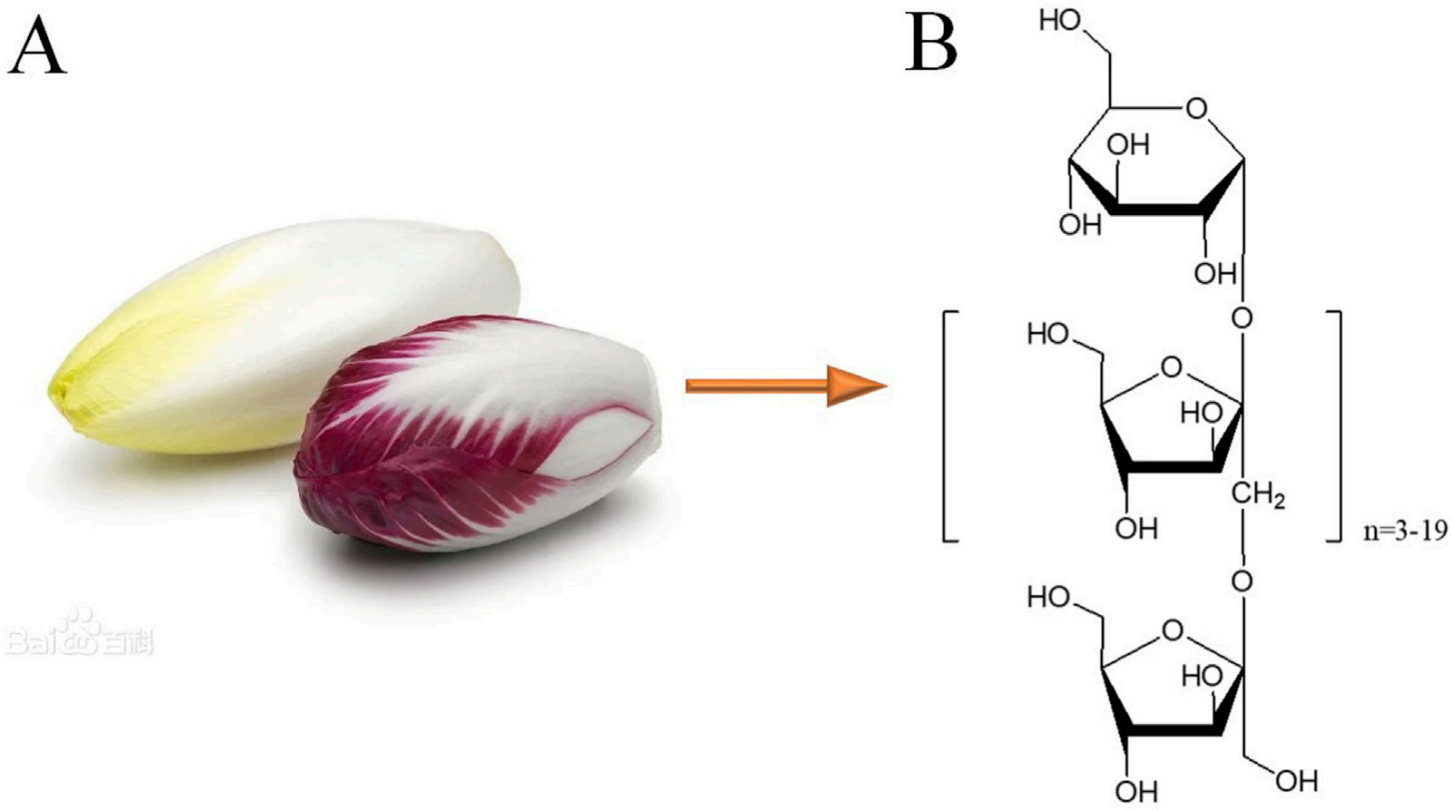
*Cichorium intybus L*. and the chemical structures of inulin-type oligosaccharides (JSO). **(A)** *Cichorium intybus L.* **(B)** Structural formula of JSO.

Inulin-type oligosaccharides (JSO) have been investigated for their potential therapeutic effects, particularly for their prebiotic properties and influence on gut-brain axis interactions. The selected doses of 50, 100, and 200 mg/kg are based on previous research that explored the dose-dependent effects of similar oligosaccharides and their impact on behavioral and physiological parameters. For instance, Burokas et al. (2017) administered different doses of fructooligosaccharides, a related compound, and found significant improvements in stress-induced behavioral deficits at doses ranging from 50 to 200 mg/kg. Sun et al. (2019) reported that administration of 100 mg/kg of a similar oligosaccharide in mice led to improved cognitive function and reduced anxiety-like behaviors. JSO could be extracted from *C. intybus L*. It was obtained from the Qingdao Tuolin Medicine Science Technology Co., Ltd. (Figure 1).

### 2.3 Grouping

Male C57BL/6N mice were randomized into six groups: control group (water treatment) (n = 12), CRS group (water treatment) (n = 12), Cit (10 mg/kg) + CRS group (n = 12), and JSO (50 mg/kg, 100 mg/kg, and 200 mg/kg, respectively) (n = 12 per group) + CRS group. The animals were orally administered with Cit, JSO, or water by gavage daily at 8. a.m. until completion of the behavioral tests. To minimize potential bias, animals were randomly assigned to each experimental group. The randomization process was conducted using a computer-generated randomization tool to ensure unbiased distribution across the control and treatment groups. In addition, all behavioral assessments and data analyses were performed by experimenters blinded to the group assignments. This double-blind approach ensured that subjective influences did not affect the outcomes of the behavioral tests, enhancing the reliability of the results. The sample size of 12 mice per group was based on power analysis and prior studies in similar models of CRS and behavioral assessments. Previous research has shown that a group size of 10–12 animals was sufficient to detect statistically significant differences in anxiety- and depression-like behaviors in rodent models, considering the variability inherent in behavioral tests (Crowley et al., 2005; Huo et al., 2017). This sample size balances the need for statistical power with ethical considerations regarding the use of animals, adhering to the principle of reduction in animal research.

### 2.4 CRS model

**FIGURE 2:**
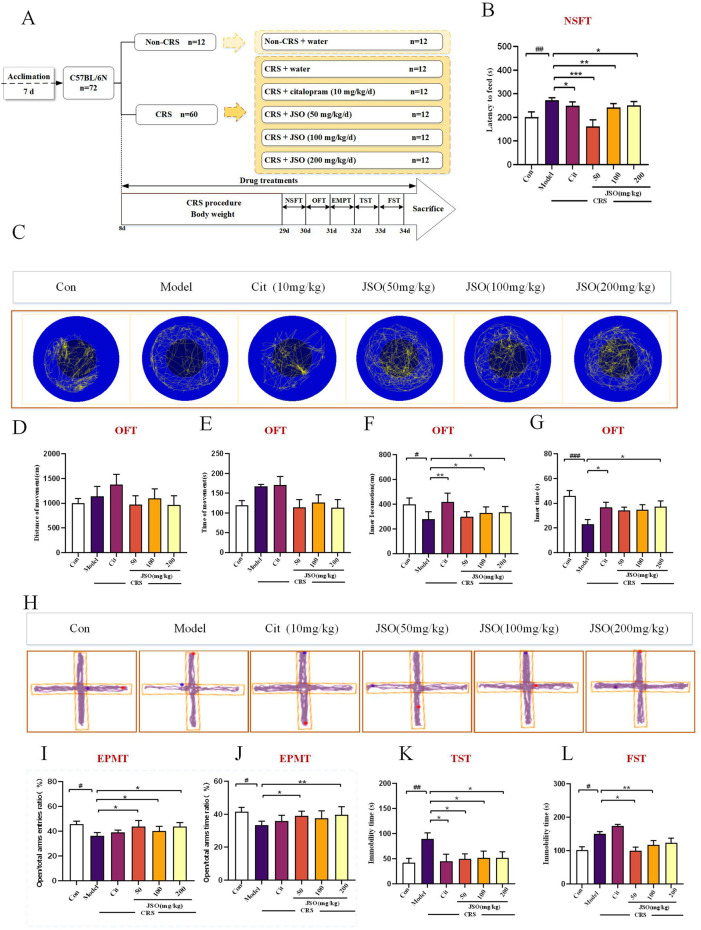
Cit and JSO can reverse anxiety/depression-like behaviors in the chronic restraint stress (CRS) mice. **(A)** Graphical representation of the procedures and drug therapeutic effects. **(B)** NSFT findings (ANOVA followed by Dunnett’s T3 post hoc test).**(C)** OFT. **(D, E)** OFT results—total distance and time travelled (ANOVA, no significant differences). **(F, G)** OFT results—distance and time in center zone (ANOVA followed by Dunnett’s T3 post hoc test). **(H)** EPMT. **(I, J)** EPMT results—open time percentage (OT%) and open entries percentage (OE%) (ANOVA followed by Dunnett’s T3 post hoc test). **(K)** TST results (ANOVA followed by Dunnett’s T3 post hoc test). **(L)** FST results (ANOVA followed by Dunnett’s T3 post hoc test). All results were presented as mean ± SEM (*n* = 8–12). ^###^ *p* < 0.001, ^##^ *p* < 0.01, and ^#^ *p* < 0.05, significantly different from the control group; **p < 0.01 and *p < 0.05, significantly different from the CRS group.

Chronic stress is a reliable method for inducing anxiety-like and depression-like behaviors in rodents, which can simulate the influence of chronic stress on human pathophysiology (Wang H. et al., 2022). The CRS model is described previously (Naert et al., 2011; Jung et al., 2013). In brief, animals were restrained in about 10 cm centrifuge tube with some small holes for 6 h every day (9:00–15:00) for 21 consecutive days. Animals were able to breathe normally but could not move around casually. During the 6 h, the animals were deprived of all food and water. The open field test (OFT), elevated plus maze test (EPMT), forced swimming test (FST), novelty-suppressed feeding test (NSFT), and tail suspension test (TST) were subsequently performed. These detailed experimental programs of the behavioral tests are shown in Figure 2A.

The oral gavage method was employed for drug administration based on its advantages in ensuring precise dosing and mimicking the oral route used in human administration. Oral gavage is a widely accepted method for administering compounds in rodent studies, as it allows for accurate control of the dose and timing of administration (Turner et al., 2011). Previous studies utilizing citalopram and oligosaccharides in rodent models have successfully employed oral gavage to achieve reliable and reproducible results (Crowley et al., 2005; Sun et al., 2019).

### 2.5 Behavioral tests

#### 2.5.1 NSFT

The NSFT is a well-established behavioral assay used to assess anxiety-like behaviors and the efficacy of potential antidepressant compounds. It measures the latency of an animal to eat in a novel, anxiety-inducing environment. Longer latencies are interpreted as increased anxiety or reduced antidepressant efficacy (Ran et al., 2018). Mice were food-deprived for 24 h before the test. The next day, the animal was moved to the NSFT room for at least 30 min. Then, the animal was placed in an open arena with food in the center. These animals were allowed to probe the novel environment for 5 min. The latency to eat, i.e., the first bite of the food, was recorded. The test arena measured 40 cm × 40 cm with 40 cm high walls. The room was illuminated with a light intensity of 50 lux. The main outcome was the discrimination index, calculated as the time spent exploring the novel object divided by the total exploration time (expressed as a percentage). Exploration times were recorded using an overhead video camera and analyzed manually with a stopwatch. Data were normalized to total exploration time and presented as mean ± standard error of the mean (SEM). Statistical analysis was performed using a paired *t*-test, and results are shown as bar graphs.

#### 2.5.2 OFT

The OFT is commonly used to evaluate general locomotor activity and anxiety-like behaviors in rodents. It assesses the willingness of an animal to explore the central, more exposed area of the open field, with reduced exploration interpreted as increased anxiety (Prut and Belzung, 2003). An open-field apparatus was used to assess locomotor activity and anxiety-like behavior. The OFT device included four white plastic tanks with a camera fixed at the top. The mice were adapted to the OFT area for <30 min before the OFT. Then, the animals were put in the center of the open field and allowed to move freely for 5 min. A reduction in the time spent and distance to the center region of the open space indicated a higher level of anxiety (Huo et al., 2017; Yoshizaki et al., 2020). The open field arena measured 50 cm × 50 cm with 40 cm high walls. The arena was illuminated with a light intensity of 100 lux at the center and 50 lux at the periphery. The main outcomes were the total distance traveled (in meters) and the time spent in the center *versus* the periphery (in seconds). Movements were tracked using an overhead video camera and analyzed with EthoVision XT software. Distance and time parameters were automatically calculated by the software. Data were log-transformed to normalize distribution and presented as mean ± SEM. Statistical analysis was done using ANOVA, and results are shown as line graphs with error bars. CRS has been demonstrated to decrease the exploration of the center of the open field, further supporting its anxiogenic effects (Kumar et al., 2015).

#### 2.5.3 EPMT

The EPMT was designed to assess anxiety-related behaviors in rodents. It measures the preference of an animal for closed, protected arms over open, exposed arms, with reduced time spent in the open arms indicating increased anxiety (Walf and Frye, 2007). The EPMT apparatus was composed of two opposite open arms and two opposite closed arms, and a center cross-platform. When testing, the animal was put at the central area of the maze toward the open arm and was allowed to probe the apparatus freely for a 5-min period. Finally, open/total arms time percentage (OT%) and open/total arm entries percentage (OE%) were calculated (Chiba et al., 2012). The maze consists of two open arms (30 cm × 5 cm) and two enclosed arms (30 cm × 5 cm × 15 cm) elevated 50 cm above the floor. The room was illuminated with a dim light of approximately 60 lux, evenly distributed across the maze. The main outcomes measured were the time spent in the open arms and the number of entries into the open arms. These parameters were recorded in seconds and counts, respectively. Data were recorded using an overhead video camera and analyzed using EthoVision XT software. Parameters were automatically assessed by the software. Data were normalized to the total exploration time and presented as mean ± SEM. Statistical analysis was performed using ANOVA, and results are shown as bar graphs. Previous studies have shown that CRS leads to a significant reduction in the time spent in the open arms, indicating increased anxiety-like behavior (Kim and Han, 2006; Buynitsky and Mostofsky, 2009).

#### 2.5.4 FST

The FST is widely utilized for evaluating depressive-like behaviors in rodents. Increased immobility time, where the animal ceases to struggle, is interpreted as a sign of behavioral despair, which is indicative of a depressive-like state. The FST is a standard assay in preclinical research for identifying potential antidepressant drugs due to its reliability and predictive validity (Cryan et al., 2002). The FST apparatus contained four plastic cylinders and with a video camera weighted at the top. The animal was forced to swim freely in an acrylic cylinder filled with four-fifths water at a temperature of 24°C ± 0.5°C for 6 min. Animals were removed 2 min before the FST, and the 4 min immobility time in the 6 min observation period was recorded (Song et al., 2020). The transparent cylinder was 20 cm in diameter and 25 cm in height, filled with water to a height of 15 cm. The room was uniformly lit with an intensity of 70 lux. The main outcome was the duration of immobility, recorded in seconds during the final 4 min of the test. Immobility time was manually assessed using a stopwatch. Data were log-transformed for normalization and presented as mean ± SEM. Statistical analysis was done using ANOVA, and results are displayed as bar graphs.

#### 2.5.5 TST

The TST is a standard assay for assessing depressive-like behaviors. Increased immobility, after an initial period of struggle, is taken as a measure of behavioral despair, and reduced immobility suggests an antidepressant-like effect. This test is based on the principle that when mice are suspended by their tails, they will initially struggle to escape, followed by a period of immobility. The duration of immobility is considered an indicator of a depressive-like state, with reduced immobility suggesting an antidepressant-like effect. The TST is a standard assay in preclinical research for identifying potential antidepressant drugs due to its high sensitivity to clinically effective antidepressants and its predictive validity (Steru et al., 1985). The TST was completed as previously described (Si et al., 2021). The mouse was suspended upside down by adhesive tape, approximately 1 cm from the tail tip and 15 cm above the ground to ensure that the animal could not climb. The animal was considered immobile when entirely motionless. The experiment continued for 6 min, and the immobility time was recorded and tested during the last 4 min. Mice were suspended using adhesive tape placed approximately 1 cm from the tip of the tail, with a suspension height of 50 cm. The room was lit with an intensity of 80 lux. The main outcome was the duration of immobility, recorded in seconds during the 6-min test. Immobility time was manually recorded with a stopwatch. Data were normalized to the total test time and presented as mean ± SEM. Statistical analysis was conducted using ANOVA, and results are presented as bar graphs.

### 2.6 Brain sample preparation

Following completion of the behavioral tests, five animals per group were anaesthetized with 10% ethyl carbamate with 0.1 M phosphate-buffered saline (PBS; pH 7.4) and containing 4% paraformaldehyde, then brain tissue samples were collected, which were fixed with 4% paraformaldehyde solution until tissue sections were obtained. The remaining mice in each group were decapitated and the brain tissue sample was immediately removed and placed in an ice bath; the medial prefrontal cortex (mPFC) and hippocampal (Hip) region were dissected and stored at −80°C.

### 2.7 Real-time quantitative polymerase chain reaction PCR (RT-qPCR)

The purity and quality of the extracted RNA were determined using a NanoDrop ND-1000 spectrophotometer (Thermo Fisher Scientific). The A260/A280 ratio was measured to assess protein contamination, with acceptable values ranging from 1.8 to 2.1. Additionally, the integrity of RNA samples was verified using an Agilent 2100 Bioanalyzer (Agilent Technologies), which provides an RNA integrity number (RIN). Samples with a RIN value above 7 were deemed suitable for further analysis. TRIzol (CW0580A; CWBIO, Beijing, China) was used to extract the total RNA from the mPFC and Hip following the instructions of the manufacturer. cDNA was synthesized using the All-in-One First-Stand cDNA Synthesis Kit (AT341-01, TransGen Biotech, Beijing, China), using 2 μg of RNA. The RT-qPCR reaction system included the TransStart Top Green qPCR SuperMix Kit at 20 mL volume and a CFX96™ Real-Time System (Bio-Rad, Beijing, China). The PCR protocol was as follows: 95°C for 10 min, followed by 40 cycles of 95°C for 15 s and 60°C for 60 s. To ensure high-quality RNA and protein samples, we followed stringent criteria before proceeding with further analyses. RNA samples were considered of acceptable quality if they met the following criteria: A260/A280 ratio between 1.8 and 2.1; RIN value above 7. Protein samples were evaluated using the Bradford assay to quantify protein concentration, ensuring consistent sample loading for subsequent analyses. Quantitative mRNA expression was performed using Bio-Rad CFX Manager 2.1. Finally, the relative mRNA expression levels were normalized to *Gapdh* between the samples and the comparative Ct method was used for data analysis (2^-△△Ct^). The qPCR primers are shown in Table 1 (Sango Biotech, Beijing, China).

**TABLE 1:** qRT-PCR primers: Il-1β, TNF-ɑ, Il-6, TGF-β, IL-10, and Gapdh (control).

Genes	Forward	Reverse
IL-1β	AAA​GCT​CTC​CAC​CTC​AAT​GG	CCC​AAG​GCC​ACA​GGT​ATT​T
TNF-ɑ	TGT​CTA​CTC​CCA​GGT​TCT​CTT	GCA​GAG​AGG​AGG​TTG​ACT​TTC
IL-6	CTT​CCA​TCC​AGT​TGC​CTT​CT	CTC​CGA​CTT​GTG​AAG​TGG​TAT​AG
TGF-β	GCT​TCT​CCC​AAG​TGT​GTC​AT	GAC​TGC​TGG​TGG​TGT​ATT​CTT
IL-10	TTG​AAT​TCC​CTG​GGT​GAG​AAG	TCC​ACT​GCC​TTG​CTC​TTA​TTT
Gapdh	AAC​AGC​AAC​TCC​CAC​TCT​TC	CCT​GTT​GCT​GTA​GCC​GTA​TT

### 2.8 Western blotting (WB)

WB was performed as previously described (Yao et al., 2023). Hip and mPFC tissues were homogenized with RIPA buffer (KeyGEN, China) to lyse the tissues and extract the protein. Total proteins extracted were quantified using the BCA protein concentration assay kit. Samples were loaded onto 8%–10% SDS-PAGE, separated using electrophoresis, and electroblotted onto nitrocellulose (NC) membranes. The NC membrane was incubated with 5% nonfat milk in TBST (Tris-buffered saline with 0.1% Tween-20) for 1.5 h at 24°C, followed by incubation overnight at 4°C with the following antibodies: c-GAS (31659S, 1:1000, Cell Signaling Technology), STING (AB288157, 1:1000, ABclone), GFAP (ab68428, 1:1000, Abcam), Iba-1 (ab178846, 1:1000, Abcam), Caspase-9 (9095S, 1:1000, Cell Signaling Technology), Caspase-8 (ab138485, 1:1000, Abcam), Bax (ab32503, 1:1000, Abcam), Bcl-2 (ab194583, 1:1000, Abcam), SYP (ab32127, 1:1000, Abcam), BDNF (A18129, 1:1000, ABclonal), Caspase-3 (ab49822, 1:1000, Abcam), NLRP3 (ab263889, 1:1000, Abcam), Caspase-1 (ab207802, 1:1000, Abcam), PSD-95 (ab18258, 1:1000, Abcam), and ASC (ab180799, 1:1000, Abcam). The next day, the NC membrane was washed three times for 10 min with TBST and incubated with HRP-labelled secondary antibodies for 1.5 h and washed thrice. The NC membranes were visualized with BeyoECL moon A and moon B (Beyotime, P0018FM), and ImageJ software was utilized to analyze bands. Images obtained were quantified using ImageJ software (NIH). Quantification was performed quantitatively by setting consistent parameters across all samples to ensure comparability. Specifically, the following steps were taken: Images were converted to 8-bit grayscale; Background subtraction was performed uniformly using the “Subtract Background” function with a rolling ball radius of 50 pixels; Regions of interest (ROIs) were defined manually for each band or stained area. The mean pixel intensity within each ROI was measured and normalized to the corresponding loading control or reference area.

### 2.9 Immunofluorescence assay

Immunofluorescence staining was completed as previously described (Machado et al., 2020; Rao et al., 2021). In brief, the whole brain tissue was placed in 4% paraformaldehyde for at least 24 h, followed by dehydration in 30% sucrose solution at 4°C. The tissue was subsequently embedded in OCT and sectioned on a freezing microtome at 30-μm-thick slices. After blocking, the slice was incubated with primary antibodies: rabbit anti-Iba1 (1:800) and rabbit anti-GFAP (1:200) at 4°C for 24 h. The sections were washed with 0.1 MPBS (5 min x 3) and before incubating for 1 h with secondary antibodies. Mounting medium solution containing DAPI was applied to mark the nuclei. Finally, the mPFC and Hip stained sections were assessed using a confocal microscope (Olympus). Images obtained were quantified using ImageJ software (NIH). Quantification was performed quantitatively by setting consistent parameters across all samples to ensure comparability. Specifically, the following steps were taken: Images were converted to 8-bit grayscale; Background subtraction was performed uniformly using the “Subtract Background” function with a rolling ball radius of 50 pixels; Regions of interest (ROIs) were defined manually for each band or stained area. The mean pixel intensity within each ROI was measured and normalized to the corresponding loading control or reference area.

### 2.10 Nissl staining

Nissl staining was performed as previously reported (Gao A. X. et al., 2023). Coronal cryosections of the fixed tissues were sectioned at 10-μm-thick and stained by a Nissl dye. The sections were washed with distilled water, soaked in 95% ethyl alcohol for 5 min, followed by dehydration. Finally, they were sealed with neutral balsam. Five slides were completed from every group. Nissl staining of the neurons was examined under an optical microscope at ×400 magnification. The value integrated optical density (IOD) of the DG region, CA1 region, and CA3 region in the Hip and mPFC were measured by ImageJ software.

### 2.11 Statistical analysis

Statistical analysis was conducted using SPSS 21.0 (IBM, Armonk, NY, USA), ImageJ, and GraphPad Prism 9.5 (GraphPad Software Inc., San Diego, CA, USA) software. All experimental results were presented as mean ± standard error of the mean (SEM). Data were analyzed using one-way analysis of variance (ANOVA), with the “model” group serving as the comparison group. The Dunnett’s T3 *post hoc* test was applied for multiple comparisons, justified due to unequal variances between groups. Outliers were identified and excluded using the Grubbs’ test, ensuring that they did not significantly influence the results. Assumptions of normality (assessed using the Shapiro-Wilk test) and homogeneity of variances (evaluated using Levene’s test) were confirmed before conducting ANOVA. A *p*-value of less than 0.05 was considered statistically significant.

## 3 Results

### 3.1 Effects of JSO on behavioral tests

To evaluate whether Cit (10 mg/kg) and JSO (50 mg/kg, 100 mg/kg, and 200 mg/kg) could alleviate CRS-induced anxiety/depression-like behaviors, we performed various behavior tests, including the NSFT, OFT, EPMT, FST, and TST (Figure 2A). The NSFT experiment was completed on day 29. CRS mice showed a significant increase in feeding latency (F_(5,50)_ = 6.417, *p < 0.01*); administration of Cit (10 mg/kg) and JSO (50, 100, and 200 mg/kg) significantly improved the feeding latency compared with the CRS group (*p < 0.05, p < 0.001, p < 0.01, p < 0.05,* respectively) (Figure 2B).

The OFT is a commonly used indicator of exploration behavior and general activity in rodents, which is used to evaluate anxiety-like behavior (Borbély et al., 2017; Dang et al., 2022). The total distance travelled and time did not show a statistically significant difference among the six groups (Figures 2D, E), indicating that CRS did not cause hyper-or hypo-locomotion. Compared with the control group, CRS markedly reduced the distance and time spent in the center zone (F (5,46) = 2.807, *p < 0.05*; F (5,52) = 3.568, *p < 0.001*) (Figures 2F, G). Notwithstanding, these decreases in the inner distances spent in the center zone were notably reversed by Cit (10 mg/kg) (*p < 0.01*) administration and JSO (100 and 200 mg/kg) administration (*p < 0.05*), and administration of Cit (10 mg/kg) (*p < 0.05*) and JSO (200 mg/kg) (*p < 0.05*) could increase inner time, demonstrating that Cit and JSO were highly effective in ameliorating CRS-induced anxiety.

In the EPMT, the OT% and OE% were used to assess anxiety-like behavior (Trujillo et al., 2021). Anxious mice were afraid to probe and stood in the closed arm of the EPMT (Zhang et al., 2017; Shanmugasundaram et al., 2020). In the EPMT, the OT% and OE% were significantly reduced in the CRS group compared with the control group (F_(5,50)_ = 1.93, *p < 0.05*; F_(5,52)_ = 2.177, *p < 0.05*). JSO significantly increased the OE% (50, 100, and 200 mg/kg) (*p < 0.05*) and OT% (50 and 200 mg/kg) (*p < 0.05* and *p < 0.01,* respectively).

Finally, CRS was shown to induce depressive-like behaviors via an increase in the immobile time in the TST and FST compared with the control group (Figures 2K, L). CRS significantly increased immobility time in both tests (FST: F_(5,56)_ = 9.477, *p < 0.05*; TST: F_(5,61)_ = 1.798, *p < 0.01*). Cit (10 mg/kg) and each dose of JSO significantly reduced immobility time in the TST (*p < 0.05*). In the FST, JSO at 50 and 100 mg/kg significantly reduced immobility time (*p < 0.01, p < 0.05,* respectively), while Cit (10 mg/kg) and JSO (200 mg/kg) exhibited no significant effect.

The results of behavioral tests suggested that JSO significantly alleviated anxiety- and depression-like behaviors induced by CRS. The lack of changes in locomotion indicates that JSO’s effects on behavior were specific to emotional regulation rather than general motor activity. These findings highlight JSO’s potential as an anxiolytic and antidepressant agent, particularly at higher doses.

### 3.2 Effects of JSO on the mRNA expression of inflammatory factors

**FIGURE 3:**
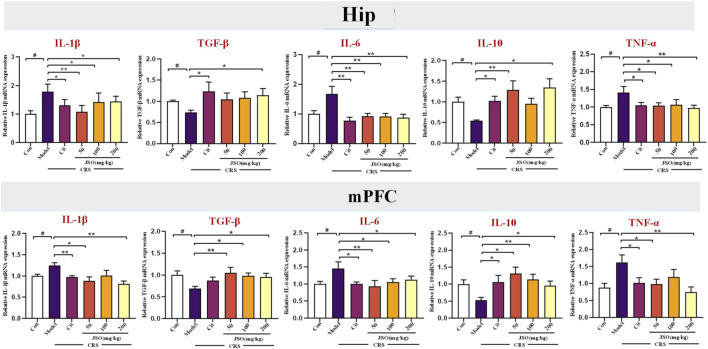
JSO treatment modulates inflammatory factor mRNA expression in the hippocampal (Hip) and medial prefrontal cortex (mPFC) regions. The histogram represents relative mRNA expression levels of inflammatory factors, including TGF-β, IL-10, IL-1β, IL-6, and TNF-α, in the Hip and mPFC regions across different groups. Statistical analysis was performed using one-way ANOVA followed by Dunnett’s T3 *post hoc* test. Data were presented as mean fold change ±SEM (n = 6–8). ^*##*^ *p < 0.01* and ^*#*^ *p < 0.05*, statistically different from the control group; ^***^ *p < 0.001,* ^**^ *p < 0.01*, and ^*^ *p < 0.05*, statistically different from the CRS group.

The histogram represents relative mRNA expression in microglial activation markers in Hip and mPFC regions in different groups (Figure 3). Compared with the sham group, significantly marked decreases were observed in the relative mRNA expression levels of TGF-β and IL-10 in the Hip (F_(5, 30)_ = 1.296, *p < 0.05*; F_(5, 32)_ = 3.556, *p < 0.05*) and mPFC (F_(5, 38)_ = 2.054, *p < 0.05*; F_(5, 41)_ = 2.2662, *p < 0.05*) regions of CRS-mice, and significant increases were observed in the relative mRNA expression levels of IL-1β, IL-6, and TNF-α in the Hip (F_(5, 37)_ = 2.710, *p < 0.05*; F_(5, 32)_ = 3.200, *p < 0.05*; F_(5, 34)_ = 2.135, *p < 0.05*) and mPFC (F_(5, 27)_ = 3.903, *p < 0.05*; F_(5, 40)_ = 2.555, *p < 0.05*; F_(5, 40)_ = 3.241, *p < 0.05*) regions of CRS-mice. Nevertheless, compared with the CRS group, Cit (10 mg/kg) and JSO (200 mg/kg) administration significantly improved the mRNA expression level of TGF-β in the Hip (*p < 0.05*), and Cit (10 mg/kg) and JSO (50 and 200 mg/kg) administration significantly improved the gene expression of IL-10 in the Hip (*p < 0.05, p < 0.01, p < 0.05,* respectively). Similarly, in the mPFC region, JSO (50, 100, and 200 mg/kg) administration markedly increased the mRNA expression level of TGF-β (*p < 0.01, p < 0.05, p < 0.05,* respectively), and Cit (10 mg/kg) and JSO (50, 100, and 200 mg/kg) administration markedly increased the mRNA expression levels of IL-10 (*p < 0.05, p < 0.05, p < 0.01, p < 0.05,* respectively). Meanwhile, Cit (10 mg/kg) and JSO (50, 100, and 200 mg/kg) administration decreased the mRNA expression level of IL-1β (*p < 0.05, p < 0.01, p < 0.05, p < 0.05,* respectively*)*, IL-6 (*p < 0.01*), and TNF-α (*p < 0.05, p < 0.05, p < 0.05, p < 0.01,* respectively) in the Hip. Moreover, Cit (10 mg/kg) and JSO (50 and 200 mg/kg) administration markedly decreased the mRNA expression levels of IL-1β (*p < 0.01, p < 0.05, p < 0.01,* respectively) and TNF-α (*p < 0.05, p < 0.05, p < 0.01,* respectively). In addition, Cit (10 mg/kg) and JSO (50, 100, and 200 mg/kg) administration markedly decreased the mRNA expression level of IL-6 (*p < 0.05, p < 0.01, p < 0.05, p < 0.05,* respectively) in the mPFC.

The modulation of expression levels of pro- and anti-inflammatory cytokines in key brain regions further supports JSO’s anti-inflammatory properties. This reduction in neuroinflammation likely contributes to the behavioral improvements observed, suggesting that JSO’s effects on anxiety and depression may be mediated through its regulation of immune responses in the brain.

### 3.3 JSO suppressed c-GAS-Sting-NLRP3 axis-mediated inflammatory activation in the hip and mPFC

**FIGURE 4:**
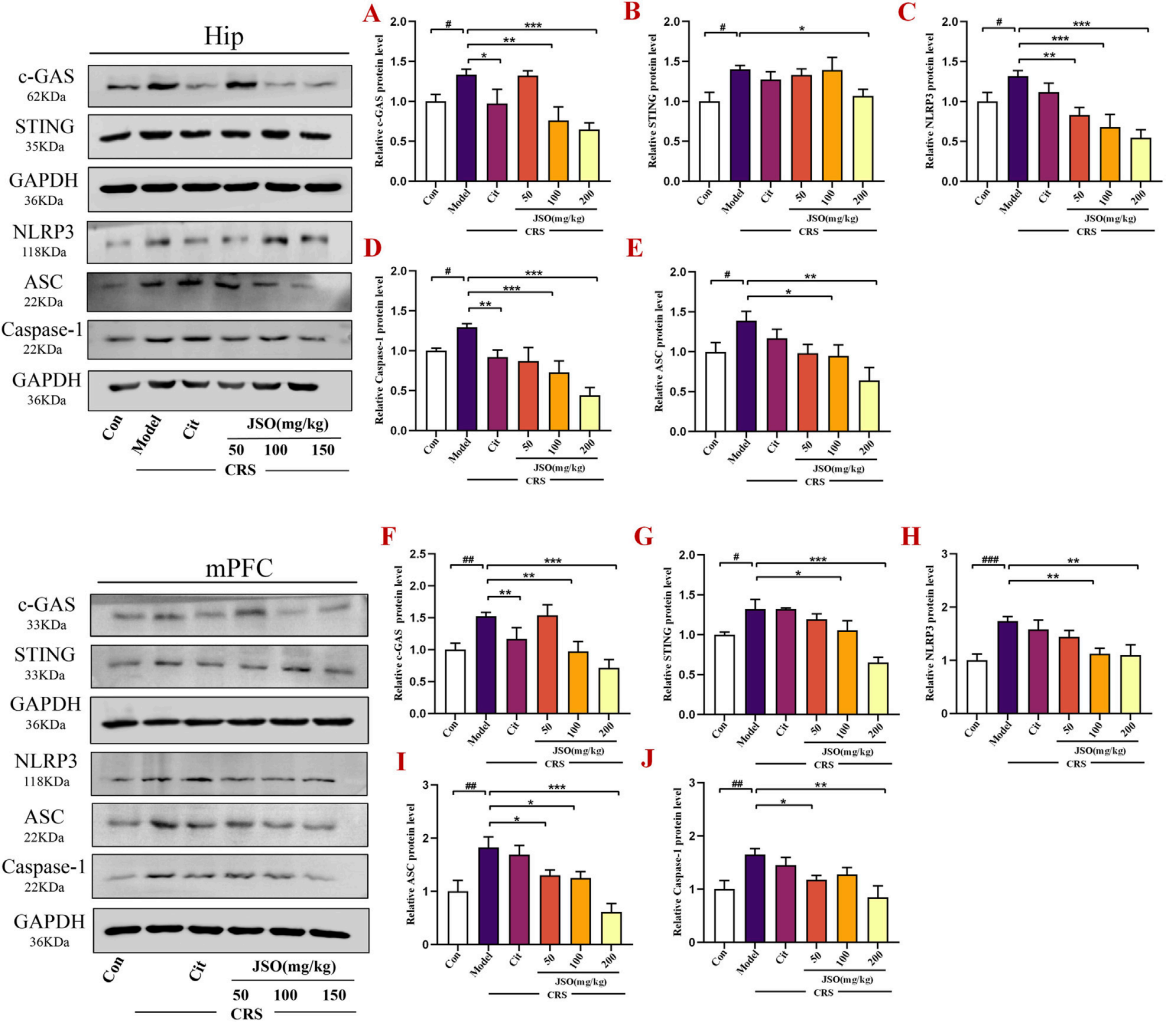
JSO suppresses c-GAS-Sting-NLRP3 axis-mediated inflammasome activation in the Hip and mPFC. **(A–E)** Protein expression levels of c-GAS, Sting, NLRP3, Caspase-1, and Asc in the Hip region. **(F–J)** Protein expression levels of c-GAS, Sting, NLRP3, Asc, and Caspase-1 in the mPFC region. Statistical analysis was performed using one-way ANOVA followed by Dunnett’s T3 *post hoc* test. Values were expressed as mean ± SEM. ^*###*^ *p < 0.001,* ^*##*^ *p < 0.01,* and ^*#*^ *p < 0.05* compared with the control group; ^**^ *p < 0.01* and ^*^ *p < 0.05* compared with the CRS group.

The NLRP3 inflammasome pathway is a multi-protein complex consisting of NLRP3, ASC, and Caspase-1, which is an important component of the inflammasome. The cGAS-STING pathway is required to activate NLRP3 in the inflammasome (Zhang et al., 2022). We assessed effects of JSO on c-GAS-Sting-NLRP3 axis-mediated inflammation in CRS-induced depression and anxiety by WB. CRS markedly increased the protein expression levels of NLRP3, Asc, Caspase-1, Sting, and c-GAS in the Hip (F_(5,29)_ = 7.533, *p < 0.05*; F_(5,14)_ = 3.301, *p < 0.05*; F_(5,21)_ = 11.520, *p < 0.05*; F_(5,12)_ = 2.855, *p < 0.05*; F_(5,22)_ = 6.399, *p < 0.05*) and in the mPFC (F_(5,25)_ = 5.418, *p < 0.001*; F_(5,12)_ = 7.496, *p < 0.01*; F_(5,12)_ = 4.109, *p < 0.01*; F_(5,13)_ = 9.997, *p < 0.05*; F_(5,12)_ = 9.049, *p < 0.01*) regions (Figure 4). Notably, Cit (10 mg/kg) and JSO (100 and 200 mg/kg) administration remarkedly downregulated protein expression levels of c-GAS (*p < 0.05, p < 0.01, p < 0.001,*respectively) and Caspase-1 (*p < 0.01, p < 0.001, p < 0.001,*respectively); JSO (200 mg/kg) reduced Sting expression level (*p < 0.05*); JSO (100 and 200 mg/kg) reduced Asc expression level (*p < 0.05, p < 0.01,* respectively); and JSO (50, 100, and 200 mg/kg) administration dramatically decreased the protein expression level of NLRP3 (*p < 0.01, p < 0.001, p < 0.001,* respectively) in the Hip region. In addition, Cit (10 mg/kg) and JSO (100 and 200 mg/kg) administration decreased c-GAS expression level (*p < 0.01, p < 0.01, p < 0.001,*respectively); JSO (100 and 200 mg/kg) administration decreased the expression levels of Sting (*p < 0.05, p < 0.001,* respectively) and NLRP3 (*p < 0.01*); JSO (50, 100, and 200 mg/kg) administration reduced Asc expression level (*p < 0.05, p < 0.05, p < 0.001,*respectively); and JSO (50 and 200 mg/kg) administration decreased Caspase-1 expression level (*p < 0.05, p < 0.01,*respectively) in the mPFC region.

The downregulation of the c-GAS-STING-NLRP3 inflammasome axis highlights JSO’s ability to target key inflammatory pathways involved in CRS-induced anxiety and depression. By suppressing this pathway, JSO may protect against neuroinflammation, explaining its beneficial effects on mood and emotional regulation.

### 3.4 Effects of JSO administration on GFAP and Iba-1 levels in the hip and mPFC

**FIGURE 5:**
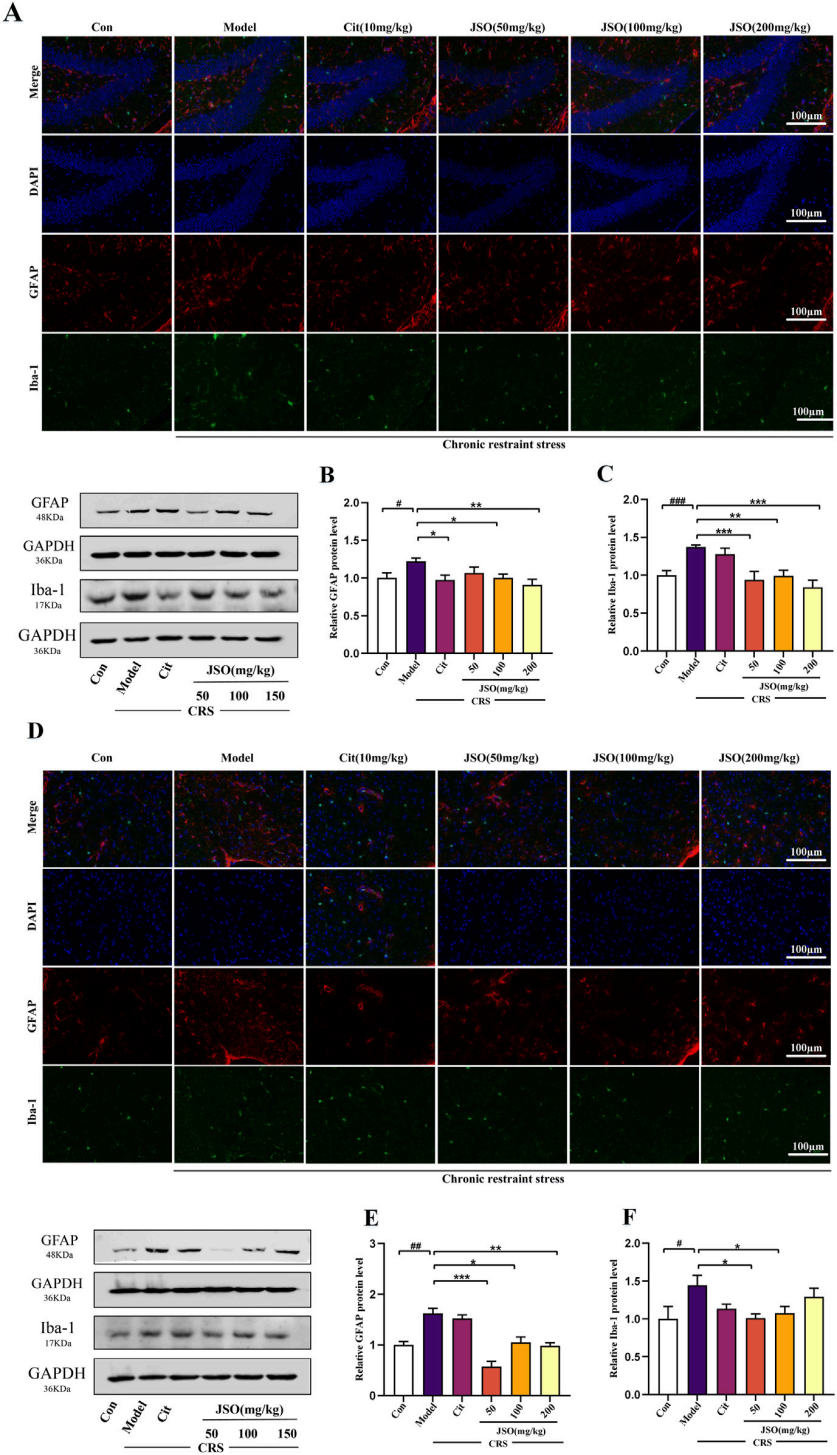
Effects of JSO on Iba-1 and GFAP expression levels in the Hip and mPFC regions. **(A)** Representative images of the GFAP and Iba-1 in the DG region of the Hip (scale bars, 100 μm). **(B, C)** GFAP and Iba-1 protein expression levels in the Hip. **(D)** Representative images of GFAP and Iba-1in the mPFC (scale bars, 100 μm). **(E, F)** GFAP and Iba-1 protein expression levels in the mPFC. Statistical analysis was performed using one-way ANOVA followed by Dunnett’s T3 post hoc test. Data were presented as mean ± SEM (n = 5). ^*###*^ *p < 0.001,* ^*##*^ *p < 0.01,* and ^*#*^ *p < 0.05*, significantly different from the control group; ^**^ *p < 0.01* and ^*^ *p < 0.05*, significantly different from the CRS group.

To further confirm CRS-induced neuroinflammation, we assessed Iba-1 and GFAP expression in the DG region of the Hip and the mPFC using immunofluorescence and WB (Figure 5). CRS increased the protein expression levels of Iba-1 and GFAP in the Hip (F_(5,18)_ = 17.506, *p < 0.001*; F_(5,29)_ = 2.865, *p < 0.05*) and mPFC (F_(5,18)_ = 2.853, *p < 0.05*; F_(5,29)_ = 16.046, *p < 0.01*); JSO (50, 100, and 200 mg/kg) significantly decreased the Iba-1 protein expression level in the Hip (*p < 0.001, p < 0.01, p < 0.001,* respectively), and GFAP expression level in the mPFC (*p < 0.001, p < 0.05, p < 0.01,* respectively). Moreover, JSO (50 and 100 mg/kg) administration significantly decreased Iba-1 expression level in the mPFC (*p < 0.05*). Similarly, Cit (10 mg/kg) and JSO (100 and 200 mg/kg) administration significantly reduced GFAP expression level in the mPFC (*p < 0.05, p < 0.05, p < 0.01,* respectively). The results further supported the hypothesis that CRS-induced neuroinflammation in the Hip and mPFC regions could be alleviated by JSO administration.

The decreased expression levels of GFAP and Iba-1 in JSO-treated mice suggests a reduction in neuroinflammation and glial activation. This decrease in neuroinflammation may be a key mechanism by which JSO ameliorates CRS-induced anxiety and depression, highlighting its neuroprotective role.

### 3.5 Effects of JSO on apoptosis-related protein expression levels in the hip and mPFC region of CRS mice

**FIGURE 6:**
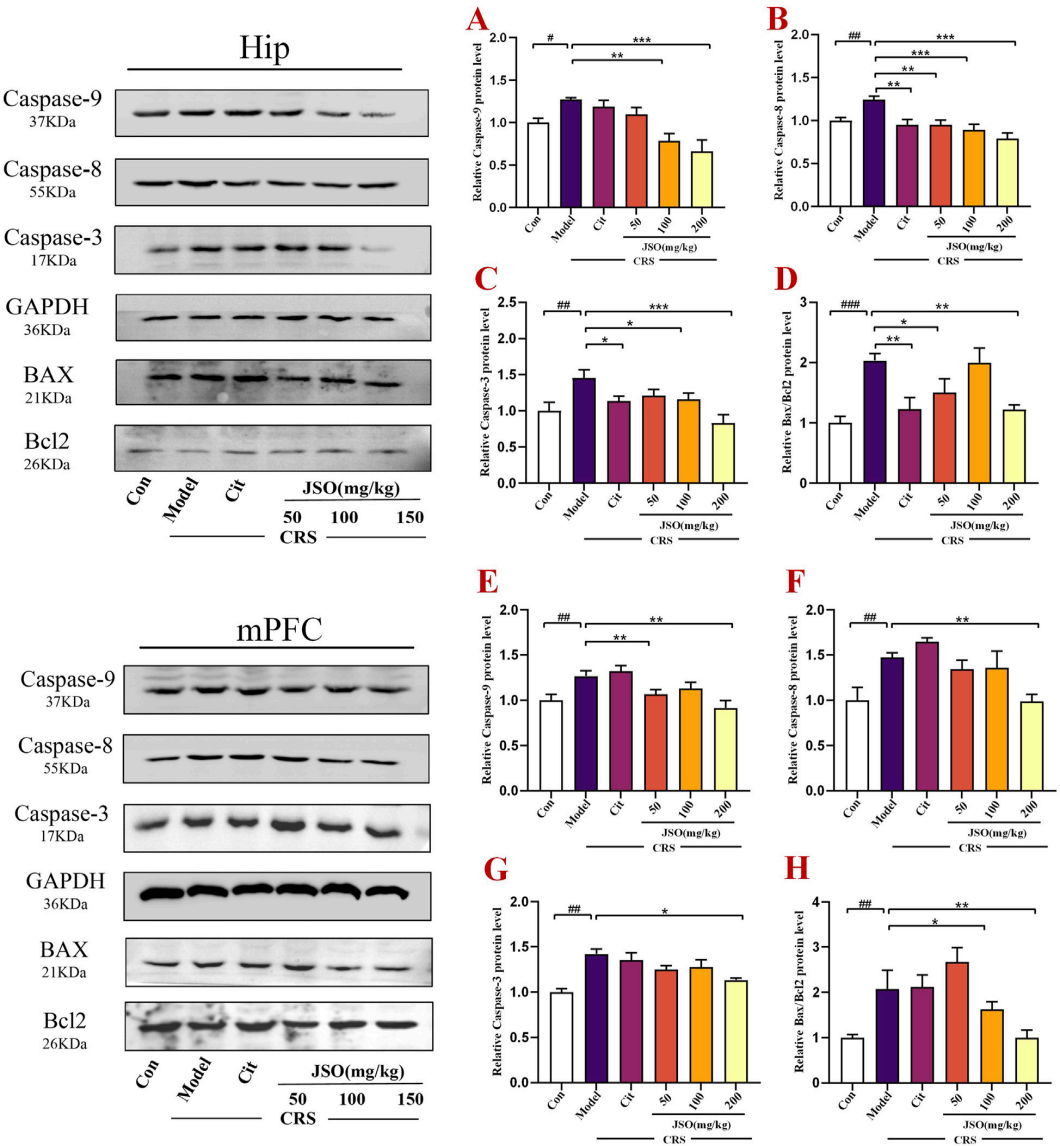
JSO administration modulates apoptosis-related proteins in the Hip and mPFC regions of CRS mice. **(A–D)** The relative protein expression levels of Caspase-3/8/9 and Bax/Bcl-2 in the Hip. **(E–H)** The relative protein expression levels of Caspase-3/8/9 and Bax/Bcl-2 in the mPFC. Statistical analysis was performed using one-way ANOVA followed by Dunnett’s T3 post hoc test. Data were presented as mean ± SEM (n = 5). ^*###*^ *p < 0.001,* ^*##*^ *p < 0.01,* and ^*#*^ *p < 0.05* compared with the control group; ^**^ *p < 0.01* and ^*^ *p < 0.05* compared with the CRS group.

Increased neuronal apoptosis has been found to be related to the anxious and depressive phenotype of CRS mice (Wang et al., 2021; Xu et al., 2023). We measured the protein expression of Bax and Bcl-2 by WB (Figure 6). Anti-apoptotic Bcl-2 expression level was reduced, whereas pro-apoptotic Bax expression level was markedly elevated in the Hip (F_(5,18)_ = 6.385, *p < 0.001*) and mPFC (F_(5,18)_ = 4.118, *p < 0.01*) regions of CRS mice. Cit (10 mg/kg) and JSO (50 and 200 mg/kg) administration increased the ratio of Bax/Bcl-2 in CRS mice compared with the control group in the Hip (*p < 0.01, p < 0.05, p < 0.01,* respectively), and the ratio of Bax/Bcl-2 was notably escalated by JSO (100 and 200 mg/kg) administration in the mPFC region (*p < 0.05, p < 0.01,* respectively). Additionally, to evaluate whether chronic stress causes apoptosis through the Caspase family, the protein expression level of Caspase-3/8/9 was analyzed in the Hip and mPFC. After exposure to CRS, Caspase-3/8/9 expression levels increased dramatically in the Hip (F_(5,36)_ = 5.358, *p < 0.01*; F_(5,42)_ = 7.643, *p < 0.01*; F_(5,18)_ = 8.259, *p < 0.05*) and mPFC (F_(5,30)_ = 9.708, *p < 0.01*; F_(5,14)_ = 6.502, *p < 0.01*; F_(5,21)_ = 6.221, *p < 0.01*). In contrast, in the Hip region, Cit (10 mg/kg) and JSO (100 and 200 mg/kg) administration substantially reduced Caspase-3 expression level (*p < 0.05, p < 0.05, p < 0.001,* respectively); Cit (10 mg/kg) and JSO (50, 100, and 200 mg/kg) markedly reduced the expression level of Caspase-8 (*p < 0.01, p < 0.01, p < 0.001, p < 0.001,* respectively); and JSO (100 and 200 mg/kg) administration reduced the expression level of Caspase-9 (*p < 0.01, p < 0.001,* respectively). Furthermore, JSO (200 mg/kg) administration significantly decreased the protein expression levels of Caspase-3 (*p < 0.05*) and Caspase-8 (*p < 0.01*), and JSO (50 and 200 mg/kg) administration markedly reduced Caspase-9 expression level (*p < 0.01*) in the mPFC.

The reduction in the expression levels of apoptosis-related proteins, particularly the Bax/Bcl-2 ratio and Caspase activity, indicated that JSO could mitigate stress-induced neuronal apoptosis. This protective effect on neurons could play a crucial role in its therapeutic effects on anxiety and depression.

### 3.6 JSO treatment significantly improved neuronal injury in the hip and mPFC

**FIGURE 7:**
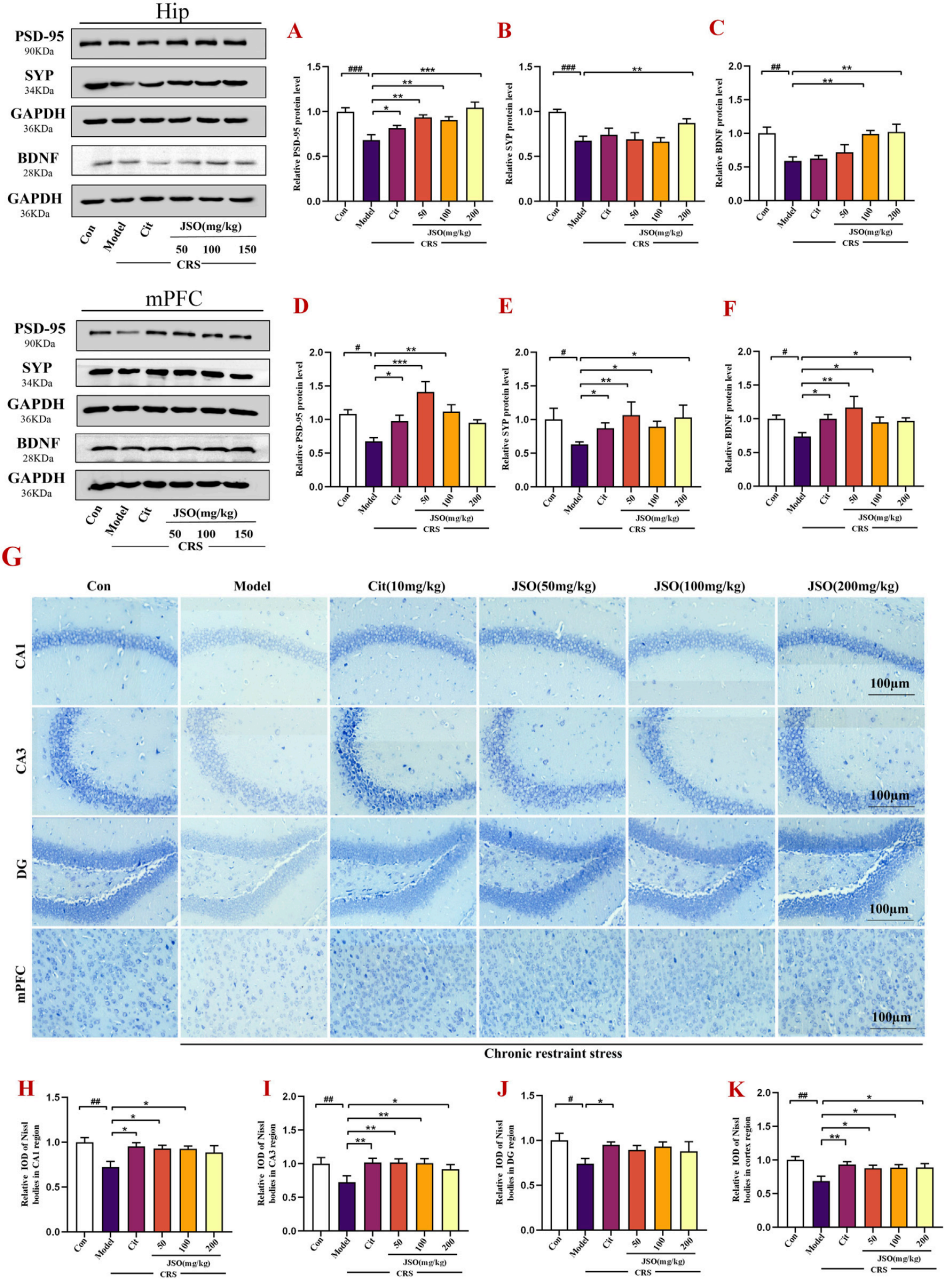
JSO treatment improves neuronal integrity in the Hip and mPFC regions. **(A–F)** Western blotting and quantification of BDNF, PSD-95, and SYP expression levels in the Hip and mPFC regions, respectively. **(G)** Nissl staining of neurons in the CA1, CA3, and DG regions of the Hip and mPFC. **(H–K)** The relative integrated optical density (IOD) values of the Nissl bodies in the Hip region and mPFC subregion. Statistical analysis was performed using one-way ANOVA followed by Dunnett’s T3 post hoc test. Data were presented as mean ± SEM (n = 5). ^*###*^ *p < 0.001,* ^*##*^ *p < 0.01,* and ^*#*^ *p < 0.05* compared with the control group; ^**^ *p < 0.01* and ^*^ *p < 0.05* compared with the CRS group.

Studies have shown that anxiety and depression are the result of neuronal dysfunction in the Hip and mPFC (Li et al., 2022). BDNF, a neurotrophic factor, is associated with the growth, differentiation, and survival of neurons (Gao et al., 2018). Furthermore, the expression of SYP and PSD-95, located on postsynaptic membrane of synapses, is closely related to synaptic plasticity (Lu et al., 2019). Therefore, we assessed the protein expression of BDNF, PSD-95 and SYP in the Hip and mPFC by WB (Figure 7). In CRS mice, the protein expression levels of BDNF, PSD-95, and SYP were significantly reduced in the Hip (F_(5,22)_ = 6.264, *p < 0.01*; F_(5,36)_ = 7.118, *p < 0.001*; F_(5,14)_ = 7.001, *p < 0.001*) and mPFC (F_(5,18)_ = 3.029, *p < 0.05;* F_(5,26)_ = 5.202, *p < 0.05*; F_(5,27)_ = 5.434, *p < 0.05*). However, Cit (10 mg/kg) and JSO (50, 100, and 200 mg/kg) administration significantly reversed the PSD-95 protein expression level in the Hip (*p < 0.05, p < 0.01, p < 0.01, p < 0.001,* respectively), and JSO (100 and 200 mg/kg) administration also markedly increased the expression levels of SYP (*p < 0.01*) and BDNF (*p < 0.01*). Similarly, in the mPFC, the expression levels of SYP (*p < 0.05, p < 0.01, p < 0.05, p < 0.05,* respectively) and BDNF (*p < 0.05, p < 0.01, p < 0.05, p < 0.05,* respectively) were elevated by Cit (10 mg/kg) and JSO (50, 100, and 200 mg/kg) administration. PSD-95 expression level (*p < 0.05, p < 0.001, p < 0.01,* respectively) increased by Cit (10 mg/kg) and JSO (50 and 100 mg/kg) administration.

Next, Nissl staining of the brain tissues was used to assess the morphological changes of the Hip and mPFC neurons. The neurons of the CA1, CA3, and DG regions of the Hip and mPFC regions were arrayed regularly and closely, and clear Nissl bodies were observed in the control group (Figure 7G). However, CRS promoted neuronal injury; the neurons were irregular and sparsely distributed, and the Nissl bodies were disintegrated. The number of Nissl-positive cells was markedly decreased in the CRS group *versus* the control group (F_(5,23)_ = 6.954, *p < 0.01*; F_(5,21)_ = 7.439, *p < 0.01*; F_(5,22)_ = 2.213, *p < 0.05*; F_(5,24)_ = 3.803, *p < 0.01*). Cit (10 mg/kg) and JSO (50 and 100 mg/kg) improved the number of Nissl-positive neurons and neuronal injury in the Hip CA1 region (*p < 0.05*); Nissl-positive neurons of the Hip CA3 region were dramatically alleviated by Cit (10 mg/kg) and JSO (50, 100, and 200 mg/kg) treatment (*p < 0.05, p < 0.01, p < 0.01, p < 0.05,* respectively); and Cit (10 mg/kg) administration increased the Nissl-positive neurons in the Hip DG (*p < 0.05*). In the mPFC region, Cit (10 mg/kg) and JSO (50, 100, and 200 mg/kg) significantly improved the disordered arrangement of neurons (*p < 0.01, p < 0.05, p < 0.05, p < 0.05,* respectively). These results showed that JSO can alleviate neuronal injury in CRS mice (Figures 7H–K).

The improvement in neuronal structure and increased expression levels of synaptic proteins, such as BDNF, PSD-95, and SYP indicated that JSO could promote neuronal health and synaptic plasticity. These effects likely contribute to its ability to reverse the detrimental impact of CRS on brain functions and emotional behaviors.

## 4 Discussion

In this study, the effects of JSO on CRS-induced behavioral deficits in mice were investigated, and related molecular alterations were explored. The administration of JSO resulted in significant behavioral improvements and changes in protein expression associated with inflammation, apoptosis, and neuroplasticity. However, it is noteworthy that while these molecular changes were observed, no pharmacological or genetic blockade was employed to confirm their causal role in the reversal of stress-induced behavioral deficits. Therefore, the findings suggested associations rather than direct causality. Future studies employing such blockade techniques are necessary to definitively establish the mechanisms by which JSO exerts its therapeutic effects.

It has recently been reported that chronic stress can induce neuroinflammation, which possibly conduces to the onset of anxiety and depression symptoms (Zhao et al., 2016; Liu et al., 2021). There is mounting evidence to suggest that chronic stress leads to the release of pro-inflammatory factors and inhibits the production of anti-inflammatory factors in the mood-related brain areas, including the Hip and mPFC regions (Huang et al., 2022; Li J. et al., 2023; Wei et al., 2023). Consistent with previous reports, the mRNA and protein expression levels of pro-inflammatory factors, including Il-1β, TNF-ɑ, and Il-6, were markedly increased in the Hip and mPFC of CRS mice. In contrast, anti-inflammatory factors, including TGF-β and IL-10, were notably reduced. Treatment with JSO significantly reversed these aforementioned changes.

Volumetric reductions in microglia and astrocytes in the Hip and PFC are one of the most documented neurological abnormalities following exposure to chronic stress (Wang Y. et al., 2022). Immunofluorescence was used to detect cell morphology in mPFC and Hip DG region. Iba-1 and GFAP are essential microglial and astrocyte markers, respectively. In activated microglia, the upregulation of Iba-1 can harm the surrounding healthy neural tissue, resulting in neuronal loss. The upregulation of GFAP is considered to be a general marker for astrogliosis (Liao W. et al., 2021). In our study, CRS induced microglial and astrocyte activation in the Hip and mPFC regions. A previous study, using an animal model showed that suppression of activated microglia could improve depressive behaviors (Zou et al., 2022). For example, minocycline treatment of rodents could prevent CRS-induced microglial expression and improve depression-like behavior (Deng et al., 2021). Astrocytes are the most abundant glial cell in the CNS and are an essential factor in the pathogenesis of psychological disorders (Liu et al., 2016). The activities of microglia and astrocytes were suppressed in the CRS mice, which was found to be assisted by the reduction of GFAP and Iba-1 expression in mPFC and Hip regions. However, JSO administration was found to significantly inhibit microglial and astrocyte activation.

Antidepressant-like effects of JSO in this study could be linked to its ability to modulate neuroinflammation, which is increasingly recognized as a central mechanism in the pathology of depression. Specifically, chronic stress-induced neuroinflammation, characterized by elevated levels of pro-inflammatory cytokines, such as IL-1β, IL-6, and TNF-α, can impair synaptic plasticity and neurotransmission, contributing to depressive symptoms (Zhang et al., 2022). JSO’s efficacy in reversing these inflammatory markers suggests that it may exert its antidepressant effects, at least in part, through mitigating neuroinflammatory responses. Although the precise molecular mechanisms underlying JSO’s antidepressant effects require further investigation, its ability to significantly suppress the c-GAS-STING-NLRP3 inflammasome pathway suggests that the compound acts by disrupting key inflammatory cascades. This is supported by the observed reduction in NLRP3 and Caspase-1 expression, both of which play a pivotal role in inflammasome activation, a process linked to depressive behaviors (Zhang et al., 2022). When comparing JSO to citalopram, a well-established SSRI, JSO’s broader modulation of both inflammatory markers and apoptosis-related proteins may point to a more complex mechanism of action. While citalopram primarily enhances serotonin availability, JSO appears to influence both neuroinflammation and neuroplasticity, as indicated by its effects on BDNF and synaptic proteins, such as PSD-95 and SYP, which are crucial for synaptic health and resilience in stress-related disorders (Gao et al., 2018). This broader impact on molecular targets could explain its similar efficacy to citalopram in ameliorating depressive symptoms despite acting through different pathways. The inflammatory hypothesis of depression highlights that elevated pro-inflammatory cytokines disrupt normal brain function and impair neurogenesis, contributing to the onset and maintenance of depressive disorders (Liu et al., 2021). JSO’s ability to significantly reduce the expression levels of IL-1β, IL-6, and TNF-α, while simultaneously elevating the levels of anti-inflammatory cytokines, such as IL-10 and TGF-β, suggesting that it plays a role in restoring the balance between pro- and anti-inflammatory signals in key mood-related regions, such as the Hip and mPFC, thereby alleviating depressive symptoms. Another important aspect of depression pathology is the impairment of neuroplasticity, which is critical for mood regulation and cognitive function (Lu et al., 2019). JSO treatment not only alleviates inflammation, but also significantly improves markers of neuroplasticity, such as BDNF, PSD-95, and SYP, suggesting that its antidepressant effects are partly due to enhancing synaptic connectivity and neuronal survival, a process that could counteract the neuronal damage induced by chronic stress.

In recent years, the cyclic GMP-AMP synthase (c-GAS)-stimulator of interferon genes (STING) signaling pathway, has been increasingly regarded as an important regulator in neuro-immunological and neurodegenerative disease, in addition to neuro-oncology. A recent study reported the activation of microglial c-GAS-STING in traumatic brain injury and neurodegenerative disease (Miyata et al., 2022; Zhang et al., 2022). Notably, in our study, we found a significant increase in the expression of c-GAS and Sting in the Hip and mPFC region of CRS mice. In addition, a previous study (Duan et al., 2022) also showed that the activated STING-TBK1-IRF3 pathway may serve as a potential mechanism to increase microglial phagocytosis by promoting the production of inflammatory factors in mice subjected to CRS, thus improving depression-like behavior (Duan et al., 2022). Previous results have shown that activated NLRP3 is the essential factor in the pathogenesis of depression and anxiety (Li S. et al., 2021). Chronic stress was reported to lead to NLRP3 inflammasome activation in the brain. Furthermore, animals showed anxiety and depression-like behaviors (Arioz et al., 2019). Suppressing the activation of the NLRP3 inflammasome exerted neuroprotective influences and improved the aforementioned behavioral changes (Li Y. et al., 2021). In our study, the protein expression of NLRP3, Asc, and Caspase-1 were activated in the mPFC and Hip regions of CRS mice, whereas JSO administration suppressed NLRP3 inflammasome activation, indicating that the anti-anxiety and anti-depressant outcome of treatment with JSO may be partly mediated via inhibition of NLRP3 inflammasome activation.

Neuroinflammation causes disturbances in the control of apoptosis; therefore, inflammatory factors are believed to be critical in promoting apoptosis in nervous system disorders (Ren et al., 2022; Xie et al., 2022). In our study, exposure to CRS markedly increased the protein Bax/Bcl2 ratio in the Hip and mPFC. Bax, as pro-apoptotic protein, can induce the production of cytochrome c into the cytosol; however, Bcl-2, as anti-apoptotic protein, can suppress the production of cytochrome c from mitochondria. Therefore, alterations in intracellular Bcl-2 and Bax expression ratios can affect mitochondrial cytochrome production. An increase in the Bax/Bcl2 ratio can induce cell apoptosis (Cheng et al., 2015; Dong et al., 2021). Pro-apoptotic family parts take part in the regulation of apoptosis. Finally, the Caspase pathways of cysteine proteases are activated, resulting in an apoptotic protease cascade response and irreversible apoptosis (Shal et al., 2019; Zhou et al., 2023) Thus, WB testing was used to explore the expression levels of apoptosis markers. We found that the protein expression of Caspase-3/8/9 in the Hip and mPFC of the CRS group was markedly increased compared to the control group. However, JSO treatment obviously improved CRS-induced apoptosis in the brain. All in all, these data suggest that JSO plays anti-anxiety and anti-depressive roles through the prevention of neuronal apoptosis.

BDNF plays an essential role in protecting and regulating the functional integrity of neurons (Li et al., 2019; Huang et al., 2023). We found that CRS exposure remarkably reduced the BDNF protein expression level in the Hip and mPFC, while treatment with JSO improved this change in CRS mice. More interestingly, the results showed that the decreased BDNF expression was accompanied by a reduction in the expression of the synaptic markers PSD-95 and SYP levels; the deficits of these synaptic proteins induced by CRS were effectively improved by JSO treatment. Nissl staining showed that the neurons in the Hip and mPFC in the CRS group were loosely arranged or missing, and Nissl bodies were lightly stained or even dissolved compared to the other groups (Dang et al., 2022; Gao Z. et al., 2023), consistent with our present results. Notably, these changes were reversed by JSO administration.

While this study provided valuable insights into the therapeutic potential of JSO in alleviating CRS-induced anxiety and depression, several limitations must be acknowledged. Firstly, although significant molecular alterations were observed, this study did not use pharmacological or genetic interventions to directly block key pathways, such as the c-GAS-STING-NLRP3 axis, to confirm their causal roles in the observed behavioral improvements. Future studies using specific inhibitors or genetic knockout models would help determine whether these pathways are directly responsible for the anti-anxiety and anti-depressant effects of JSO. Secondly, the study focused on molecular changes in the Hip and mPFC, while other brain regions implicated in stress-related disorders, such as the amygdala or nucleus accumbens, were not explored. Future research will expand the investigation to these areas to provide a more comprehensive understanding of JSO’s mechanisms of action across different brain regions. Finally, another notable limitation is the lack of investigation into the prebiotic activity of JSO and its potential effects on gut microbiota and the gut-brain axis. Future studies should incorporate microbiota analysis and explore how changes in gut microbiota may influence the behavioral and molecular outcomes observed. Additionally, future research will explore the long-term effects of JSO treatment to determine its potential for sustained therapeutic benefits and assess any possible side effects or toxicities over extended periods.

## 5 Conclusion

The results indicated that JSO has potential therapeutic effects on stress-induced behavioral deficits in a CRS mouse model, as evidenced by behavioral improvements and associated molecular changes. However, further research is needed to confirm the causal mechanisms underlying these effects.

## Data Availability

The original contributions presented in the study are included in the article/Supplementary Material, further inquiries can be directed to the corresponding authors.

## References

[B1] An L. Yang J. C. Yin H. Xue R. Wang Q. Sun Y. C. (2016). Inulin-type oligosaccharides extracted from yacon produce antidepressant-like effects in behavioral models of depression. Phytotherapy Res. PTR 30 (12), 1937–1942. 10.1002/ptr.5698 27539187

[B2] Arioz B. I. Tastan B. Tarakcioglu E. Tufekci K. U. Olcum M. Ersoy N. (2019). Melatonin attenuates LPS-induced acute depressive-like behaviors and microglial NLRP3 inflammasome activation through the SIRT1/nrf2 pathway. Front. Immunol. 10, 1511. 10.3389/fimmu.2019.01511 31327964 PMC6615259

[B3] Borbély É. Hajna Z. Nabi L. Scheich B. Tékus V. László K. (2017). Hemokinin-1 mediates anxiolytic and anti-depressant-like actions in mice. Brain, Behav. Immun. 59, 219–232. 10.1016/j.bbi.2016.09.004 27621226

[B85] Burokas A. Arboleya S. Moloney R. D. Peterson V. L. Murphy K. Clarke G. (2017). Targeting the microbiota-gut-brain axis: prebiotics have anxiolytic and antidepressant-like effects and reverse the impact of chronic stress in mice. Biol Psychiatry. 82 (7), 472–487. 10.1016/j.biopsych.2016.12.031 28242013

[B5] Buynitsky T. Mostofsky D. I. (2009). Restraint stress in biobehavioral research: recent developments. Neurosci. and Biobehav. Rev. 33 (7), 1089–1098. 10.1016/j.neubiorev.2009.05.004 19463853

[B6] Caetano B. F. R. de Moura N. A. Almeida A. P. S. Dias M. C. Sivieri K. Barbisan L. F. (2016). Yacon (Smallanthus sonchifolius) as a food supplement: health-promoting benefits of fructooligosaccharides. Nutrients 8 (7), 436. 10.3390/nu8070436 27455312 PMC4963912

[B7] Chen Z. Gu J. Lin S. Xu Z. Xu H. Zhao J. (2023). Saffron essential oil ameliorates CUMS-induced depression-like behavior in mice via the MAPK-CREB1-BDNF signaling pathway. J. Ethnopharmacol. 300, 115719. 10.1016/j.jep.2022.115719 36126781

[B8] Cheng C. H. Yang F. F. Ling R. Z. Liao S. A. Miao Y. T. Ye C. X. (2015). Effects of ammonia exposure on apoptosis, oxidative stress and immune response in pufferfish (Takifugu obscurus). Aquat. Toxicol. Amst. Neth. 164, 61–71. 10.1016/j.aquatox.2015.04.004 25917764

[B9] Chiba S. Numakawa T. Ninomiya M. Richards M. C. Wakabayashi C. Kunugi H. (2012). Chronic restraint stress causes anxiety- and depression-like behaviors, downregulates glucocorticoid receptor expression, and attenuates glutamate release induced by brain-derived neurotrophic factor in the prefrontal cortex. Prog. Neuro-psychopharmacology and Biol. Psychiatry 39 (1), 112–119. 10.1016/j.pnpbp.2012.05.018 22664354

[B10] Crowley J. J. Blendy J. A. Lucki I. (2005). Strain-dependent antidepressant-like effects of citalopram in the mouse tail suspension test. Psychopharmacol. Berl. 183 (2), 257–264. 10.1007/s00213-005-0166-5 16220334

[B4] Crowley J. J. Brodkin E. S. Blendy J. A. Berrettini W. H. Lucki I. (2006). Antidepressant-like effects of citalopram in the tail suspension test in mice. Neuropsychopharmacology 31 (11), 2433–2442. 10.1038/sj.npp.1301065 16554742

[B11] Cryan J. F. Markou A. Lucki I. (2002). Assessing antidepressant activity in rodents: recent developments and future needs. Trends Pharmacol. Sci. 23 (5), 238–245. 10.1016/s0165-6147(02)02017-5 12008002

[B12] Dang R. Wang M. Li X. Wang H. Liu L. Wu Q. (2022). Edaravone ameliorates depressive and anxiety-like behaviors via Sirt1/Nrf2/HO-1/Gpx4 pathway. J. Neuroinflammation 19 (1), 41. 10.1186/s12974-022-02400-6 35130906 PMC8822843

[B13] Deng I. Corrigan F. Garg S. Zhou X.-F. Bobrovskaya L. (2021). Further characterization of intrastriatal lipopolysaccharide model of Parkinson's disease in C57bl/6 mice. Int. J. Mol. Sci. 22 (14), 7380. 10.3390/ijms22147380 34299000 PMC8304722

[B14] Dong X. Lu K. Lin P. Che H. Li H. Song L. (2021). Saccharina japonica ethanol extract ameliorates depression/anxiety-like behavior by inhibiting inflammation, oxidative stress, and apoptosis in dextran sodium sulfate induced ulcerative colitis mice. Front. Nutr. 8, 784532. 10.3389/fnut.2021.784532 34977127 PMC8716690

[B15] Duan N. Zhang Y. Tan S. Sun J. Ye M. Gao H. (2022). Therapeutic targeting of STING-TBK1-IRF3 signalling ameliorates chronic stress induced depression-like behaviours by modulating neuroinflammation and microglia phagocytosis. Neurobiol. Dis. 169, 105739. 10.1016/j.nbd.2022.105739 35470042

[B16] Fan J. Guo F. Mo R. Chen L.-Y. Mo J.-W. Lu C.-L. (2023). O-GlcNAc transferase in astrocytes modulates depression-related stress susceptibility through glutamatergic synaptic transmission. J. Clin. Investigation 133 (7), e160016. 10.1172/JCI160016 PMC10065078 36757814

[B17] Fu Q. Qiu R. Chen L. Chen Y. Qi W. Cheng Y. (2023). Music prevents stress-induced depression and anxiety-like behavior in mice. Transl. Psychiatry 13 (1), 317. 10.1038/s41398-023-02606-z 37828015 PMC10570293

[B18] Gao A. X. Xia T. C. X. Peng Z. T. Wu Q. Y. Zhu Y. Dong T. T. X. (2023a). The ethanolic extract of peanut shell attenuates the depressive-like behaviors of mice through modulation of inflammation and gut microbiota. Food Res. Int. Ott. Ont. 168, 112765. 10.1016/j.foodres.2023.112765 37120215

[B19] Gao J. Li K. Du L. Yin H. Tan X. Yang Z. (2018). Deletion of asparagine endopeptidase reduces anxiety- and depressive-like behaviors and improves abilities of spatial cognition in mice. Brain Res. Bull. 142, 147–155. 10.1016/j.brainresbull.2018.07.010 30030107

[B20] Gao Z. Lv H. Wang Y. Xie Y. Guan M. Xu Y. (2023b). TET2 deficiency promotes anxiety and depression-like behaviors by activating NLRP3/IL-1β pathway in microglia of allergic rhinitis mice. Mol. Med. Camb. Mass 29 (1), 160. 10.1186/s10020-023-00757-9 38012545 PMC10680276

[B21] Grippo A. J. Francis J. Beltz T. G. Felder R. B. Johnson A. K. (2005). Neuroendocrine and cytokine profile of chronic mild stress-induced anhedonia. Physiology and Behav. 84 (5), 697–706. 10.1016/j.physbeh.2005.02.011 15885245

[B22] Huang C. Zhang F. Li P. Song C. (2022). Low-dose IL-2 attenuated depression-like behaviors and pathological changes through restoring the balances between IL-6 and TGF-β and between Th17 and treg in a chronic stress-induced mouse model of depression. Int. J. Mol. Sci. 23 (22), 13856. 10.3390/ijms232213856 36430328 PMC9699071

[B23] Huang J. Huang W. Yi J. Deng Y. Li R. Chen J. (2023). Mesenchymal stromal cells alleviate depressive and anxiety-like behaviors via a lung vagal-to-brain axis in male mice. Nat. Commun. 14 (1), 7406. 10.1038/s41467-023-43150-0 37973914 PMC10654509

[B24] Huo R. Zeng B. Zeng L. Cheng K. Li B. Luo Y. (2017). Microbiota modulate anxiety-like behavior and endocrine abnormalities in hypothalamic-pituitary-adrenal Axis. Front. Cell. Infect. Microbiol. 7, 489. 10.3389/fcimb.2017.00489 29250490 PMC5715198

[B25] Jung J. M. Park S. J. Lee Y. W. Lee H. E. Hong S. I. Lew J. H. (2013). The effects of a standardized Acanthopanax koreanum extract on stress-induced behavioral alterations in mice. J. Ethnopharmacol. 148 (3), 826–834. 10.1016/j.jep.2013.05.019 23721913

[B26] Kim K. S. Han P. L. (2006). Optimization of chronic stress paradigms using anxiety- and depression-like behavioral parameters. J. Neurosci. Res. 83 (3), 497–507. 10.1002/jnr.20754 16416425

[B27] Kumar A. Singh A. Ekavali (2015). A review on Alzheimer's disease pathophysiology and its management: an update. Pharmacol. Rep. 67 (2), 195–203. 10.1016/j.pharep.2014.09.004 25712639

[B28] Kwatra M. Ahmed S. Gangipangi V. K. Panda S. R. Gupta N. Shantanu P. A. (2021). Lipopolysaccharide exacerbates chronic restraint stress-induced neurobehavioral deficits: mechanisms by redox imbalance, ASK1-related apoptosis, autophagic dysregulation. J. Psychiatric Res. 144, 462–482. 10.1016/j.jpsychires.2021.10.021 34768069

[B29] Lau T. Bigio B. Zelli D. McEwen B. S. Nasca C. (2017). Stress-induced structural plasticity of medial amygdala stellate neurons and rapid prevention by a candidate antidepressant. Mol. Psychiatry 22 (2), 227–234. 10.1038/mp.2016.68 27240534 PMC5133196

[B30] Letenneur V. Monnoye M. Philippe C. Holowacz S. Rabot S. Lepage P. (2023). Effects of a lacticaseibacillus mix on behavioural, biochemical, and gut microbial outcomes of male mice following chronic restraint stress. Nutrients 15 (21), 4635. 10.3390/nu15214635 37960288 PMC10648220

[B31] Li J. Wang H. Liu D. Li X. He L. Pan J. (2023a). CB2R activation ameliorates late adolescent chronic alcohol exposure-induced anxiety-like behaviors during withdrawal by preventing morphological changes and suppressing NLRP3 inflammasome activation in prefrontal cortex microglia in mice. Brain, Behav. Immun. 110, 60–79. 10.1016/j.bbi.2023.02.001 36754245

[B32] Li S. Sun Y. Song M. Song Y. Fang Y. Zhang Q. (2021a). NLRP3/caspase-1/GSDMD-mediated pyroptosis exerts a crucial role in astrocyte pathological injury in mouse model of depression. JCI Insight 6 (23), e146852. 10.1172/jci.insight.146852 34877938 PMC8675200

[B33] Li W. Y. Gao J. Y. Lin S. Y. Pan S. T. Xiao B. Ma Y. T. (2022). Effects of involuntary and voluntary exercise in combination with acousto-optic stimulation on adult neurogenesis in an alzheimer's mouse model. Mol. Neurobiol. 59 (5), 3254–3279. 10.1007/s12035-022-02784-9 35297012

[B34] Li X. Zhang J. Niu R. Manthari R. K. Yang K. Wang J. (2019). Effect of fluoride exposure on anxiety- and depression-like behavior in mouse. Chemosphere 215, 454–460. 10.1016/j.chemosphere.2018.10.070 30336322

[B35] Li Y. Song W. Tong Y. Zhang X. Zhao J. Gao X. (2021b). Isoliquiritin ameliorates depression by suppressing NLRP3-mediated pyroptosis via miRNA-27a/SYK/NF-κB axis. J. Neuroinflammation 18 (1), 1. 10.1186/s12974-020-02040-8 33402173 PMC7786465

[B36] Li Y. Yang L. Li J. Gao W. Zhao Z. Dong K. (2023b). Antidepression of Xingpijieyu formula targets gut microbiota derived from depressive disorder. CNS Neurosci. and Ther. 29 (2), 669–681. 10.1111/cns.14049 36550591 PMC9873506

[B37] Liao M. Zhang Y. Qiu Y. Wu Z. Zhong Z. Zeng X. (2021b). Fructooligosaccharide supplementation alleviated the pathological immune response and prevented the impairment of intestinal barrier in DSS-induced acute colitis mice. Food and Funct. 12 (20), 9844–9854. 10.1039/d1fo01147b 34664584

[B38] Liao W. Liu Y. Wang L. Cai X. Xie H. Yi F. (2021a). Chronic mild stress-induced protein dysregulations correlated with susceptibility and resiliency to depression or anxiety revealed by quantitative proteomics of the rat prefrontal cortex. Transl. Psychiatry 11 (1), 143. 10.1038/s41398-021-01267-0 33627638 PMC7904772

[B78] Lightowler H. Thondre S. Holz A. Theis S. (2018). Replacement of glycaemic carbohydrates by inulin-type fructans from chicory (oligofructose, inulin) reduces the postprandial blood glucose and insulin response to foods: report of two double-blind, randomized, controlled trials. Eur. J. Nutr. 57 (3), 1259–1268. 10.1007/s00394-017-1409-z 28255654

[B39] Liu B. Zhang Y. Yang Z. Liu M. Zhang C. Zhao Y. (2021). ω-3 DPA protected neurons from neuroinflammation by balancing microglia M1/M2 polarizations through inhibiting NF-κB/MAPK p38 signaling and activating neuron-BDNF-PI3K/AKT pathways. Mar. Drugs 19 (11), 587. 10.3390/md19110587 34822458 PMC8619469

[B40] Liu J. Dupree J. L. Gacias M. Frawley R. Sikder T. Naik P. (2016). Clemastine enhances myelination in the prefrontal cortex and rescues behavioral changes in socially isolated mice. J. Neurosci. Official J. Soc. Neurosci. 36 (3), 957–962. 10.1523/JNEUROSCI.3608-15.2016 PMC4719024 26791223

[B41] Liu W. Z. Zhang W. H. Zheng Z. H. Zou J. X. Liu X. X. Huang S. H. (2020). Identification of a prefrontal cortex-to-amygdala pathway for chronic stress-induced anxiety. Nat. Commun. 11 (1), 2221. 10.1038/s41467-020-15920-7 32376858 PMC7203160

[B42] Lu Y. Sun G. Yang F. Guan Z. Zhang Z. Zhao J. (2019). Baicalin regulates depression behavior in mice exposed to chronic mild stress via the Rac/LIMK/cofilin pathway. Biomed. and Pharmacother. = Biomedecine and Pharmacother. 116, 109054. 10.1016/j.biopha.2019.109054 31176122

[B43] Ma J. Wang R. Chen Y. Wang Z. Dong Y. (2023). 5-HT attenuates chronic stress-induced cognitive impairment in mice through intestinal flora disruption. J. Neuroinflammation 20 (1), 23. 10.1186/s12974-023-02693-1 36737776 PMC9896737

[B44] MacDowell K. S. Martín-Hernández D. Ulecia-Morón C. Bris Á. G. Madrigal J. L. M. García-Bueno B. (2021). Paliperidone attenuates chronic stress-induced changes in the expression of inflammasomes-related protein in the frontal cortex of male rats. Int. Immunopharmacol. 90, 107217. 10.1016/j.intimp.2020.107217 33290967

[B45] Machado D. G. Lara M. V. S. Dobler P. B. Almeida R. F. Porciúncula L. O. (2020). Caffeine prevents neurodegeneration and behavioral alterations in a mice model of agitated depression. Prog. Neuro-psychopharmacology and Biol. Psychiatry 98, 109776. 10.1016/j.pnpbp.2019.109776 31707092

[B46] Miyata S. Ishino Y. Shimizu S. Tohyama M. (2022). Involvement of inflammatory responses in the brain to the onset of major depressive disorder due to stress exposure. Front. Aging Neurosci. 14, 934346. 10.3389/fnagi.2022.934346 35936767 PMC9354609

[B47] Monteiro S. Roque S. de Sá-Calçada D. Sousa N. Correia-Neves M. Cerqueira J. J. (2015). An efficient chronic unpredictable stress protocol to induce stress-related responses in C57BL/6 mice. Front. Psychiatry 6, 6. 10.3389/fpsyt.2015.00006 25698978 PMC4313595

[B48] Moreira Szokalo R. A. Redko F. Ulloa J. Flor S. Tulino M. S. Muschietti L. (2020). Toxicogenetic evaluation of Smallanthus sonchifolius (yacon) as a herbal medicine. J. Ethnopharmacol. 257, 112854. 10.1016/j.jep.2020.112854 32325177

[B49] Morel C. Montgomery S. E. Li L. Durand-de Cuttoli R. Teichman E. M. Juarez B. (2022). Midbrain projection to the basolateral amygdala encodes anxiety-like but not depression-like behaviors. Nat. Commun. 13 (1), 1532. 10.1038/s41467-022-29155-1 35318315 PMC8940900

[B50] Naert G. Ixart G. Maurice T. Tapia-Arancibia L. Givalois L. (2011). Brain-derived neurotrophic factor and hypothalamic-pituitary-adrenal axis adaptation processes in a depressive-like state induced by chronic restraint stress. Mol. Cell. Neurosci. 46 (1), 55–66. 10.1016/j.mcn.2010.08.006 20708081

[B80] Perović J. Tumbas Šaponjac V. Kojić J. Krulj J. Moreno D. A. García-Viguera C. (2021). Chicory (*Cichorium intybus* L.) as a food ingredient—Nutritional composition, bioactivity, safety, and health claims: A review. Food Chem 30, 127676. 10.1016/j.foodchem.2020.127676 32768902

[B81] Prut L. Belzung C. (2003). The open field as a paradigm to measure the effects of drugs on anxiety-like behaviors: a review. Eur. J. Pharmacol. 463 (1–3), 3–33. 10.1016/s0014-2999(03)01272-x 12600700

[B51] Ran Y. H. Hu X. X. Wang Y. L. Zhao N. Zhang L. M. Liu H. X. (2018). YL-0919, a dual 5-HT1A partial agonist and SSRI, produces antidepressant- and anxiolytic-like effects in rats subjected to chronic unpredictable stress. Acta Pharmacol. Sin. 39 (1), 12–23. 10.1038/aps.2017.83 28858297 PMC5758671

[B52] Rao J. Qiao Y. Xie R. Lin L. Jiang J. Wang C. (2021). Fecal microbiota transplantation ameliorates stress-induced depression-like behaviors associated with the inhibition of glial and NLRP3 inflammasome in rat brain. J. Psychiatric Res. 137, 147–157. 10.1016/j.jpsychires.2021.02.057 33677218

[B53] Ren Y. Sun Waterhouse D. Ouyang F. Tan X. Li D. Xu L. (2022). Apple phenolic extracts ameliorate lead-induced cognitive impairment and depression- and anxiety-like behavior in mice by abating oxidative stress, inflammation and apoptosis via the miR-22-3p/SIRT1 axis. Food and Funct. 13 (5), 2647–2661. 10.1039/d1fo03750a 35167638

[B54] Shal B. Khan A. Naveed M. Ullah Khan N. Ihsan Ul H. D AlSharari S. (2019). Effect of 25-methoxy hispidol A isolated from Poncirus trifoliate against bacteria-induced anxiety and depression by targeting neuroinflammation, oxidative stress and apoptosis in mice. Biomed. and Pharmacother. = Biomedecine and Pharmacother. 111, 209–223. 10.1016/j.biopha.2018.12.047 30583228

[B55] Shanmugasundaram J. Subramanian V. Nadipelly J. Kathirvelu P. Sayeli V. Cheriyan B. V. (2020). Anxiolytic-like activity of 5-methoxyflavone in mice with involvement of GABAergic and serotonergic systems - *in vivo* and *in silico* evidences. Eur. Neuropsychopharmacol. J. Eur. Coll. Neuropsychopharmacol. 36, 100–110. 10.1016/j.euroneuro.2020.05.009 32534819

[B56] Shen F. Song Z. Xie P. Li L. Wang B. Peng D. (2021). Polygonatum sibiricum polysaccharide prevents depression-like behaviors by reducing oxidative stress, inflammation, and cellular and synaptic damage. J. Ethnopharmacol. 275, 114164. 10.1016/j.jep.2021.114164 33932516

[B57] Si L. Wang Y. Liu M. Yang L. Zhang L. (2021). Expression and role of microRNA-212/nuclear factor I-A in depressive mice. Bioengineered 12 (2), 11520–11532. 10.1080/21655979.2021.2009964 34889698 PMC8810195

[B58] Song A. Q. Gao B. Fan J. J. Zhu Y. J. Zhou J. Wang Y. L. (2020). NLRP1 inflammasome contributes to chronic stress-induced depressive-like behaviors in mice. J. Neuroinflammation 17 (1), 178. 10.1186/s12974-020-01848-8 32513185 PMC7281929

[B59] Steru L. Chermat R. Thierry B. Simon P. (1985). The tail suspension test: a new method for screening antidepressants in mice. Psychopharmacol. Berl. 85 (3), 367–370. 10.1007/BF00428203 3923523

[B60] Sun J. Liu S. Ling Z. Wang F. Ling Y. Gong T. (2019). Fructooligosaccharides ameliorating cognitive deficits and neurodegeneration in APP/PS1 transgenic mice through modulating gut microbiota. J. Agric. Food Chem. 67, 3006–3017. 10.1021/acs.jafc.8b07313 30816709

[B61] Trujillo V. Valentim Lima E. Mencalha R. Carbalan Q. S. R. Dos-Santos R. C. Felintro V. (2021). Neonatal serotonin depletion induces hyperactivity and anxiolytic-like sex-dependent effects in adult rats. Mol. Neurobiol. 58 (3), 1036–1051. 10.1007/s12035-020-02181-0 33083963

[B62] Turner P. V. Brabb T. Pekow C. Vasbinder M. A. (2011). Administration of substances to laboratory animals: routes of administration and factors to consider. J. Am. Assoc. Laboratory Animal Sci. 50 (5), 600–613. PMC3189662 22330705

[B63] Ulrich-Lai Y. M. Herman J. P. (2009). Neural regulation of endocrine and autonomic stress responses. Nat. Rev. Neurosci. 10 (6), 397–409. 10.1038/nrn2647 19469025 PMC4240627

[B64] Walf A. A. Frye C. A. (2007). The use of the elevated plus maze as an assay of anxiety-related behavior in rodents. Nat. Protoc. 2 (2), 322–328. 10.1038/nprot.2007.44 17406592 PMC3623971

[B65] Wang H. Huang H. Jiang N. Zhang Y. Lv J. Liu X. (2022a). Tenuifolin ameliorates chronic restraint stress-induced cognitive impairment in C57BL/6J mice. Phytotherapy Res. PTR 36 (3), 1402–1412. 10.1002/ptr.7402 35129236

[B66] Wang H. Jiang N. Lv J. Huang H. Liu X. (2020). Ginsenoside Rd reverses cognitive deficits by modulating BDNF-dependent CREB pathway in chronic restraint stress mice. Life Sci. 258, 118107. 10.1016/j.lfs.2020.118107 32682919

[B67] Wang X. Wang Z. Cao J. Dong Y. Chen Y. (2021). Melatonin ameliorates anxiety-like behaviors induced by sleep deprivation in mice: role of oxidative stress, neuroinflammation, autophagy and apoptosis. Brain Res. Bull. 174, 161–172. 10.1016/j.brainresbull.2021.06.010 34144202

[B68] Wang Y. Wu Z. Chen H. Liu R. Zhang W. Chen X. (2022b). Astragalus polysaccharides protect against inactivated Vibrio alginolyticus-induced inflammatory injury in macrophages of large yellow croaker. Fish and Shellfish Immunol. 131, 95–104. 10.1016/j.fsi.2022.09.077 36206995

[B69] Wang Y.-L. Han Q.-Q. Gong W.-Q. Pan D.-H. Wang L.-Z. Hu W. (2018). Microglial activation mediates chronic mild stress-induced depressive- and anxiety-like behavior in adult rats. J. Neuroinflammation 15 (1), 21. 10.1186/s12974-018-1054-3 29343269 PMC5773028

[B70] Wei H. Yu C. Zhang C. Ren Y. Guo L. Wang T. (2023). Butyrate ameliorates chronic alcoholic central nervous damage by suppressing microglia-mediated neuroinflammation and modulating the microbiome-gut-brain axis. Biomed. and Pharmacother. = Biomedecine and Pharmacother. 160, 114308. 10.1016/j.biopha.2023.114308 36709599

[B71] Xie J. Liu L. Guo H. Bao Q. Hu P. Li H. (2022). Orally administered melanin from Sepiapharaonis ink ameliorates depression-anxiety-like behaviors in DSS-induced colitis by mediating inflammation pathway and regulating apoptosis. Int. Immunopharmacol. 106, 108625. 10.1016/j.intimp.2022.108625 35180627

[B72] Xu D.-H. Du J.-K. Liu S.-Y. Zhang H. Yang L. Zhu X.-Y. (2023). Upregulation of KLK8 contributes to CUMS-induced hippocampal neuronal apoptosis by cleaving NCAM1. Cell Death and Dis. 14 (4), 278. 10.1038/s41419-023-05800-5 PMC10115824 37076499

[B73] Yao C. Zhang Y. Sun X. Pei H. Wei S. Wang M. (2023). Areca catechu L. ameliorates chronic unpredictable mild stress-induced depression behavior in rats by the promotion of the BDNF signaling pathway. Biomed. and Pharmacother. = Biomedecine and Pharmacother. 164, 114459. 10.1016/j.biopha.2023.114459 37245336

[B74] Yoshizaki K. Asai M. Hara T. (2020). High-fat diet enhances working memory in the Y-maze test in male C57bl/6J mice with less anxiety in the elevated plus maze test. Nutrients 12 (7), 2036. 10.3390/nu12072036 32659954 PMC7400900

[B75] Zhang C. Song Y. Chen L. Chen P. Yuan M. Meng Y. (2022). Urolithin A attenuates hyperuricemic nephropathy in fructose-fed mice by impairing STING-NLRP3 axis-mediated inflammatory response via restoration of parkin-dependent mitophagy. Front. Pharmacol. 13, 907209. 10.3389/fphar.2022.907209 35784701 PMC9240289

[B76] Zhang M. Liu Y. Zhao M. Tang W. Wang X. Dong Z. (2017). Depression and anxiety behaviour in a rat model of chronic migraine. J. Headache Pain 18 (1), 27. 10.1186/s10194-017-0736-z 28224378 PMC5319946

[B77] Zhou Y. Ren W. Sun Q. Yu K. M. Lang X. Li Z. (2021). The association of clinical correlates, metabolic parameters, and thyroid hormones with suicide attempts in first-episode and drug-naïve patients with major depressive disorder comorbid with anxiety: a large-scale cross-sectional study. Transl. Psychiatry 11 (1), 97. 10.1038/s41398-021-01234-9 33542178 PMC7862235

[B84] Zhou M. Fan Y. Xu L. Yu Z. Wang S. Xu H. (2023). Microbiome and tryptophan metabolomics analysis in adolescent depression: roles of the gut microbiota in the regulation of tryptophan-derived neurotransmitters and behaviors in human and mice. Microbiome 11 (1), 145. 10.1186/s40168-023-01589-9 37386523 PMC10311725

[B82] Zou J. Shang W. Yang L. Liu T. Wang L. Li X. (2022). Microglia activation in the mPFC mediates anxiety-like behaviors caused by Staphylococcus aureus strain USA300. Brain Behav. 12 (9), e2715. 10.1002/brb3.2715 35977050 PMC9480961

[B83] Zhao Q. Wu X. Yan S. Xie X. Fan Y. Zhang J. (2016). The antidepressant-like effects of pioglitazone in a chronic mild stress mouse model are associated with PPARγ-mediated alteration of microglial activation phenotypes. J. Neuroinflammation 13 (1), 259. 10.1186/s12974-016-0728-y 27716270 PMC5051050

