# Supplementary material for: Effects of inulin-type oligosaccharides (JSO) from Cichorium intybus L. on behavioral deficits induced by chronic restraint stress in mice and associated molecular alterations
Source: Front Pharmacol. 2024 Nov 1;15:1484337. doi: 10.3389/fphar.2024.1484337 (PMC11563967; doi:10.3389/fphar.2024.1484337)

Supplementary Materials

Figure 1. Original western blot for three repeats of hippocampus


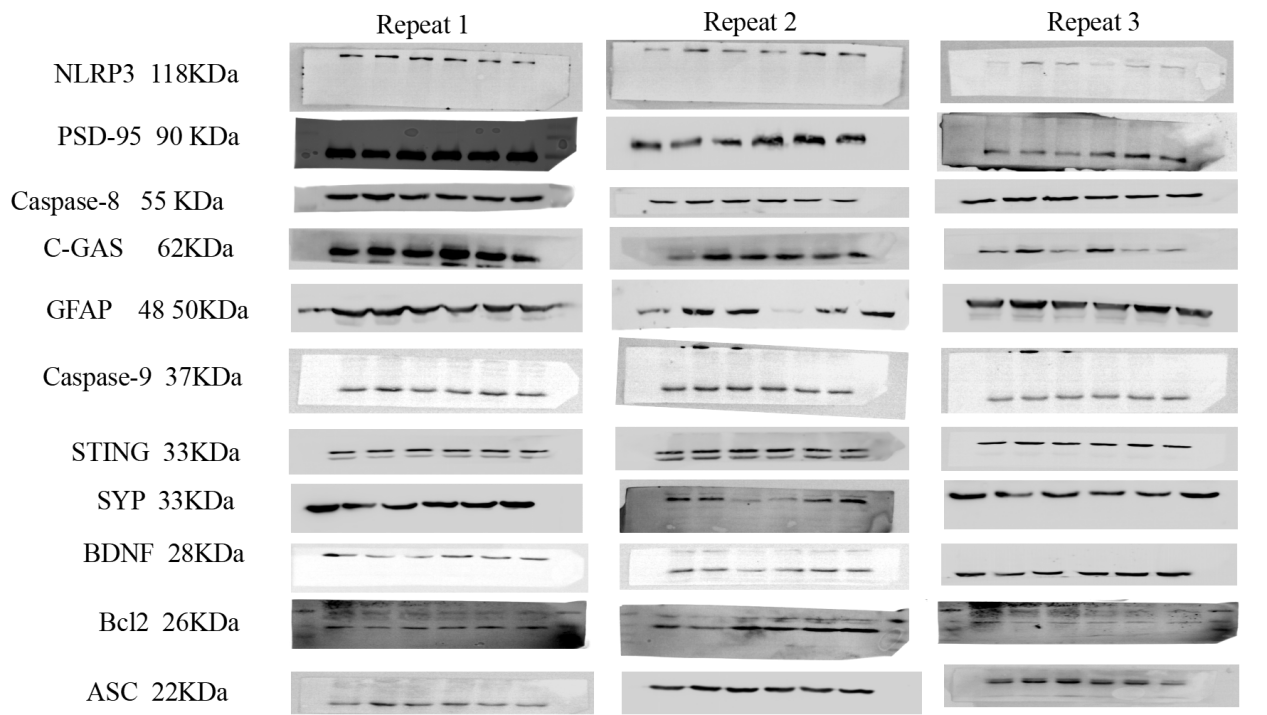


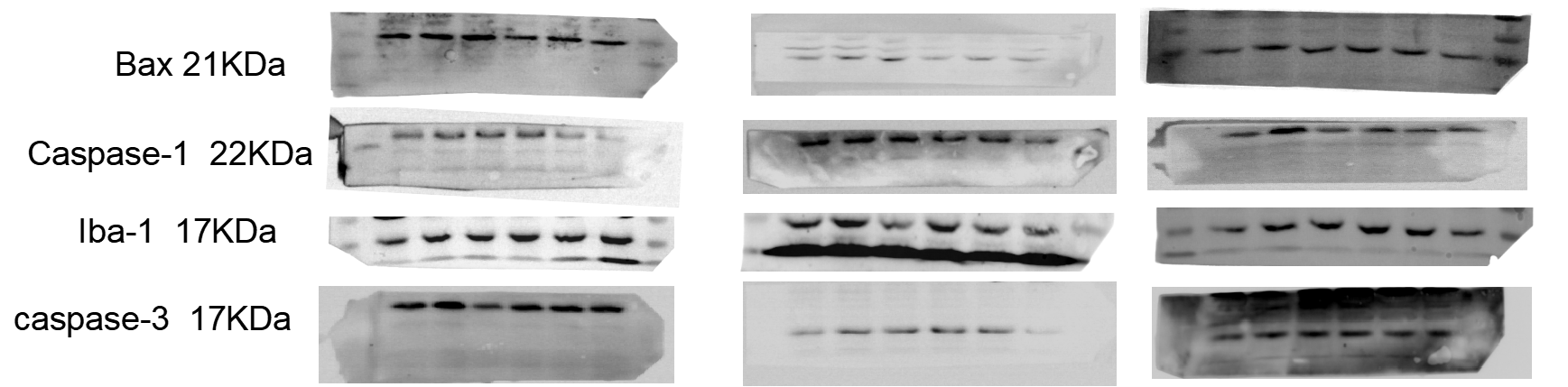

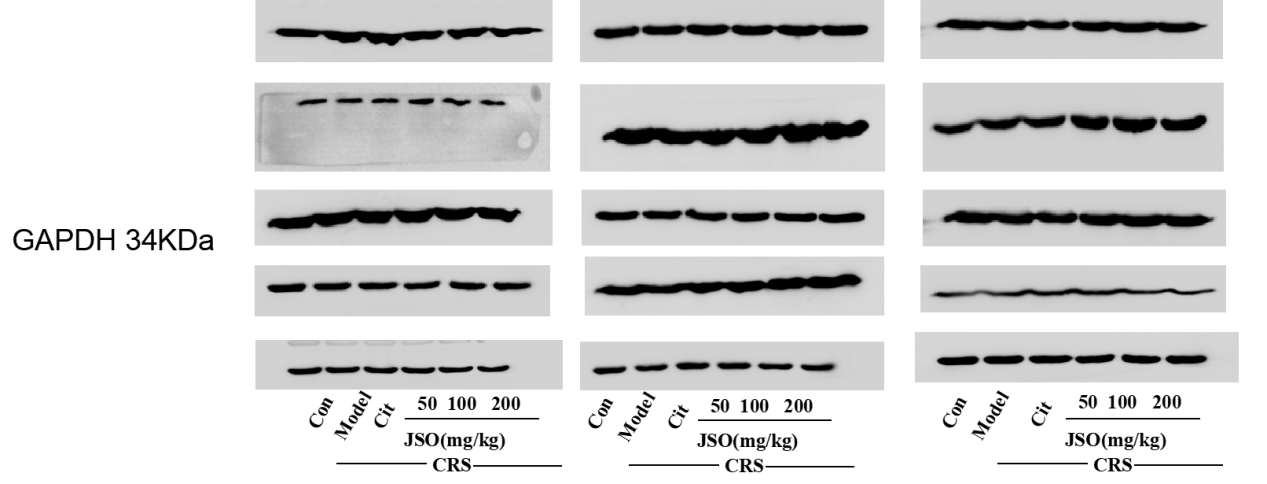


Figure 2. Original western blot for three repeats of medial prefrontal cortex


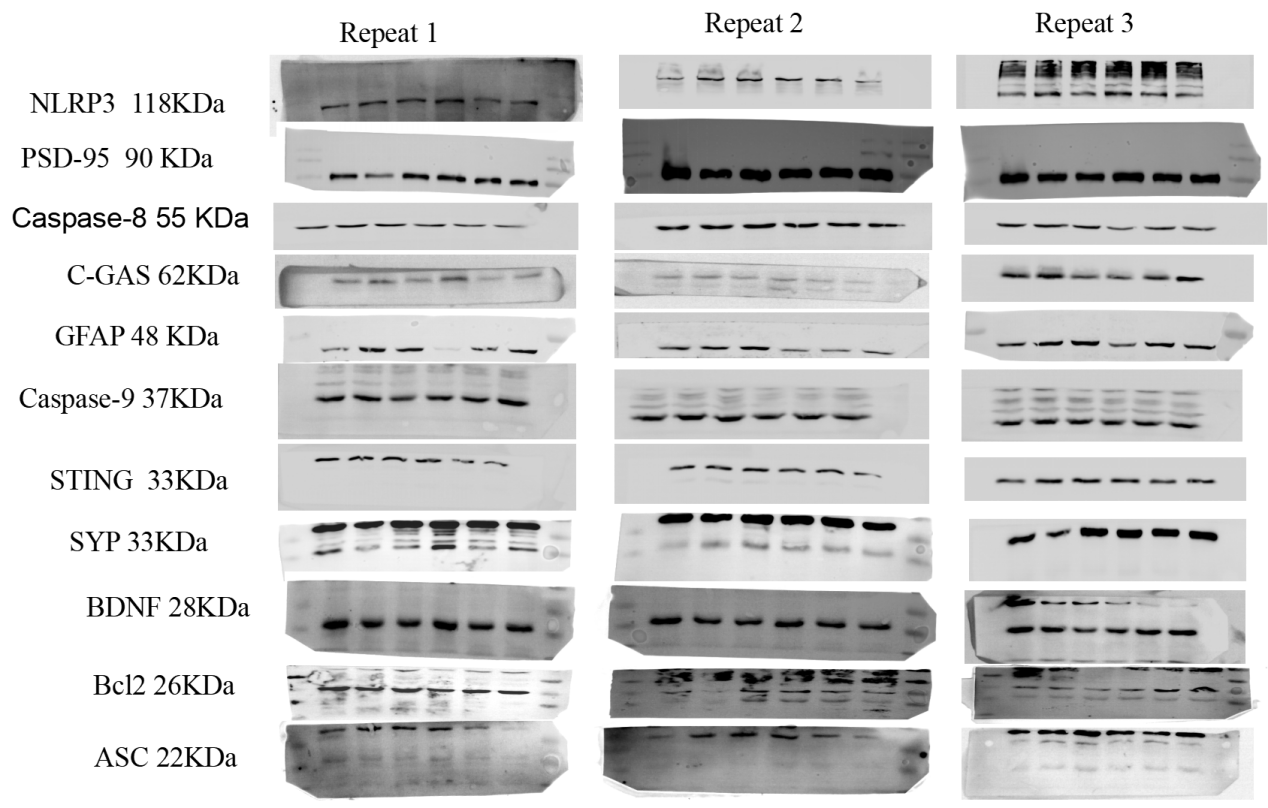

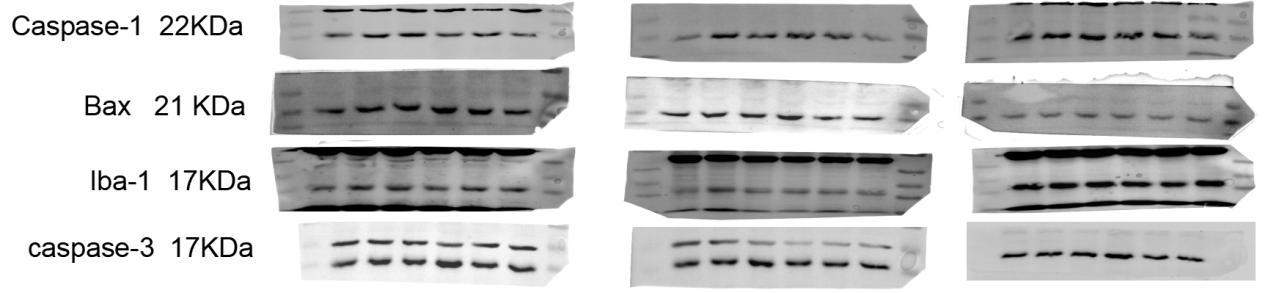

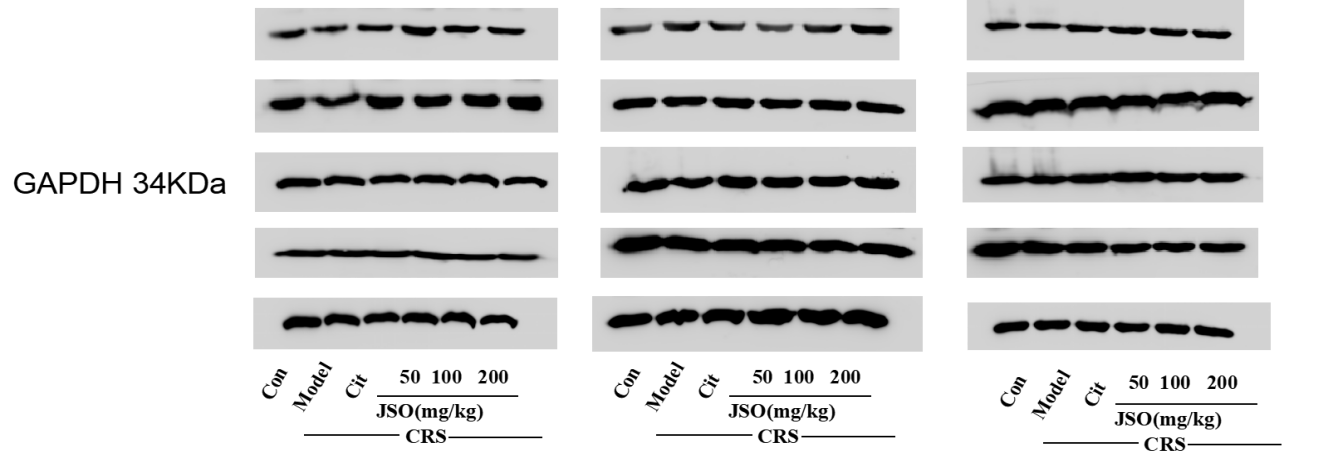

Supplement: Supplementary file 1 [file DataSheet1.ZIP › Supplementary Materials.docx]
